# Serum metabolomic profiling reveals load-specific adaptations to resistance training

**DOI:** 10.1007/s11306-026-02514-5

**Published:** 2026-08-23

**Authors:** Diego Salgueiro, Matthews Martins, Guilherme Scherrer, Denis Valério, Alex Castro, Renato Barroso, Richard Leite, Valerio Barauna

**Affiliations:** 1 School of Physical Education and Sport of Ribeirão Preto, University of São Paulo, Ribeirão Preto, Brazil; 2 Department of Physiological Sciences, Federal University of Espírito Santo, Vitória, Brazil; 3 Laboratory of Strength and Conditioning, LAFEC, Center for Physical Education and Sports, Federal University of Espírito Santo, Vitória, Brazil; 4 Universidade Estadual de Campinas, School of Physical Education, Department of Sport Sciences, Campinas, Brazil; 5 National Institute of Science and Technology - INCT Physical (In)activity and Health, CNPq, Brasília, Brazil; 6 Brazilian Biosciences National Laboratory (LNBio), Brazilian Center for Research in Energy and Materials (CNPEM), São Paulo, Brazil

**Keywords:** Resistance training, Metabolomics, Random forest, Load-specific, Branched-chain amino acid catabolism

## Abstract

**Introduction:**

Resistance training (RT) imposes repeated mechanical stimuli triggering systemic metabolic reprogramming whose molecular underpinnings remain incompletely characterized. Univariate approaches fail to capture pathway-level shifts defining the metabolomic response to RT in protocols differing in load.

**Objectives:**

To evaluate whether high-load (HL) and low-load (LL) RT protocols performed to volitional failure induce serum biochemical patterns specific to training status and loading condition, undetectable by univariate approaches.

**Methods:**

Seventeen healthy young men completed an 8-week RT intervention (HL: 80% 1-RM, n = 9; LL: 30% 1-RM, n = 8). Fasting serum profiles were acquired by untargeted ^1^H-NMR spectroscopy. Univariate comparisons used paired t-tests and one-way ANOVA with Benjamini–Hochberg correction. Random Forest models were validated by stratified 5 × 5-fold cross-validation and 1000-iteration permutation testing, with group separation assessed by sensitivity, specificity, and AUC. Discriminant metabolites were independently mapped onto established metabolic pathways to assess biochemical plausibility.

**Results:**

Of 10 metabolites significantly altered, five were consistently modulated across both protocols (3-hydroxyisovalerate, 3-hydroxybutyrate, acetone, isobutyrate, and lactate), reflecting shared adaptations in amino acid turnover and ketone body metabolism. Training-status separation achieved AUC = 1.00 (p < 0.001), with 3-hydroxyisovalerate as the dominant feature. Load-specific divergence was captured only by an exploratory multivariate signature of choline, glucose, and alanine (AUC = 0.94; p = 0.008), none of which was individually significant in univariate testing. Pathway integration demonstrated that discriminant metabolites are consistently related to well-established metabolic pathways: leucine catabolism, ketone body turnover, and glycolytic–oxidative rebalancing.

**Conclusions:**

RT induces biologically coherent, load-modulated serum metabolomic shifts detectable only through multivariate analysis. These findings are hypothesis-generating and require external validation in independent cohorts before applied implementation.

**Supplementary Information:**

The online version contains supplementary material available at https://doi.org/10.1007/s11306-026-02514-5.

## Introduction

Resistance training (RT) generates a cascade of metabolic intermediates that act as systemic signaling molecules, coordinating adaptive responses across different metabolic networks (Baker & Rutter, 2023; Curovic, 2025; Furrer & Handschin, 2024; Furrer et al., 2023). These circulating metabolites represent a dynamic molecular snapshot of the adaptive state, shaped not only by training but also by specific characteristics, including training status and the magnitude of the mechanical load applied (Morville et al., 2020). Although some investigations have employed methods to identify adaptations between different forms of RT training (Brunelli et al., 2019; Furrer & Handschin, 2024; González-Alcázar et al., 2025; Jenkins et al., 2017; Schoenfeld et al., 2017), few have specifically examined the load-dependent serum metabolomic signatures of HL and LL protocols. Consequently, the molecular signatures distinguishing high-load (HL; ≥ 70% 1-RM) from low-load (LL; ≤ 35% 1-RM) resistance training remain poorly defined (Yeom et al., 2023).

This gap is notable given that both protocols, when performed to volitional failure, produce equivalent gains in muscle hypertrophy and strength (Jenkins et al., 2017; Schoenfeld et al., 2017), yet likely engage partially divergent intracellular signaling cascades (Jenkins et al., 2015; Ozaki et al., 2016). These diverging responses can also be explained by other biochemical pathway activation or, as seen for greater oxygen consumption and enduring lipolysis in LL compared to HL training (Grisebach et al., 2024). However, the current literature is limited in terms of comparability because it often involves different protocols in subjects, such as clinical groups (Kambic et al., 2021), combines aerobic training (Kambič, 2023), or involves models of blood flow restriction (Centner et al., 2019; Haddock et al., 2021; Kamiş et al., 2024). We still do not fully understand the fingerprints in the blood induced by HL versus LL training, and whether some combinations of metabolites can reliably indicate the status, type, and intensity of training remains unclear.

A core analytical challenge is that the metabolic response to RT is fundamentally multivariate and pathway-embedded, yet conventional univariate approaches cannot exploit this integrative nature, remaining blind to the covariance patterns that encode coordinated metabolic information invisible to single-metabolite testing. Indeed, univariate analyses of high-load and low-load RT performed to volitional failure in healthy men have failed to reveal the coordinated pathway-level shifts that characterize distinct loading and training-status conditions (Valério et al., 2023). Multivariate analysis of the serum metabolome addresses this limitation directly, simultaneously capturing the integrated output of multiple interconnected metabolic pathways (Kell & Oliver, 2016). This divergence between univariate and multivariate outcomes is itself a well-described feature of metabolomic data: information absent at the level of individual metabolites can emerge from the correlation structure among them when they are analyzed jointly (Saccenti et al., 2014).

In this regard, the present work addresses a specific and previously unresolved question: could multivariate analysis reveal subtle chronic signatures when high- and low-load RT converge on equivalent hypertrophic and strength gains, even when no individual metabolite reaches significance in the direct univariate comparison between both protocols? Its contribution rests on three distinct elements. (i) The novel object is the chronic serum metabolomic signature distinguishing the two loading conditions—a contrast that, as outlined above, has been examined at the level of neuromuscular and intramuscular signaling but not in the circulating metabolome. (ii) The multivariate framework is the means of resolving this contrast, which univariate testing captures only a limited fraction of the information (Valério et al., 2023). (iii) The pathway-level interpretation is the step at which this signature acquires physiological meaning, turning a statistical separation into a biologically interpretable account of RT adaptation.

## Methods

### Participants

Seventeen healthy young men (age 24 ± 3 years, height 173 ± 6 cm, and body mass 71 ± 13.7 kg) participated in an 8-week resistance training program and were divided into two groups: high-load (HL; n = 9, age 24 ± 3 years, height 173 ± 6 cm, and body weight 72 ± 14 kg) and low-load (LL; n = 8, age 23 ± 4 years, height 172 ± 7 cm, and body weight 69 ± 13 kg). Participants received information about the benefits, risks, and experimental procedures involved in the study, and signed an informed consent form before participation. The study was conducted in accordance with the Declaration of Helsinki and approved by the University’s Ethics Committee (CAAE 47086115.4.0000.5404). Further methodological details are available in a previous publication by our group (Valério et al., 2023).

### 8-Week resistance training program

All participants underwent familiarization and 1-RM testing for leg press (45°) and leg extension exercises prior to the intervention. Participants completed two weekly training sessions in the following order: leg press at 45° and bilateral leg extension exercises, over 8 weeks. Each session began with a general warm-up (5 min on a stationary bicycle) and a specific warm-up (one set of 10 repetitions at 50% of 1-RM), followed by three sets at 80% of 1-RM in the HL group and 30% in the LL group. Both groups performed repetitions until concentric fatigue, with 90 s rest between sets and 120 s rest between exercises. Concentric fatigue was defined as the point at which the participants could no longer complete another repetition within a specified range of motion. Loads were reassessed mid-intervention. Blood samples were collected at two timepoints, before to the first and last training sessions, following a 10-h overnight fast and 1 h of seated rest upon arrival at the laboratory. At both timepoints, participants consumed a standardized meal (290 kcal; 60% carbohydrates, 25% lipids, 15% proteins) one hour before blood collection, ensuring equivalent pre-analytical metabolic conditions across sampling occasions.

### Blood metabolome via ^1^H-NMR

Serum samples (350 µL) were filtered through pre-washed 3 kDa centrifugal filters (Amicon Ultra, Sigma-Aldrich, St. Louis, MO, USA) by centrifugation at 14,000 rpm for 45 min at 4 °C. The resulting filtrate (200 µL) was mixed with 60 µL of 0.1 mol/L phosphate buffer (NaH_2_PO_4_/Na_2_HPO_4_, pH 7.4), 6.06 µL of 50 mmol/L TMSP-d_4_ in D_2_O (internal chemical shift reference), and 340 µL of Milli-Q water. The final solution was transferred to 5-mm NMR tubes (Wilmad-LabGlass, Vineland, NJ, USA) for spectral acquisition (Valério et al., 2023).

^1^H-NMR spectra were acquired on a 600 MHz Agilent spectrometer (Agilent Technologies Inc., Santa Clara, CA, USA) at 298 K using 256 scans, a 1.5 s relaxation delay, and a 4 s acquisition time. Spectra were processed in Chenomx NMR Suite 7.6 (Chenomx Inc., Edmonton, Canada), including manual phase and baseline correction, exclusion of the residual water resonance (4.6–5.1 ppm), application of 0.3 Hz line broadening, and chemical shift referencing to TMSP-d_4_ at δ = 0.00 ± 0.001 ppm. Metabolites were identified and quantified using the Chenomx targeted profiling library.

Spectral quality was assessed using predefined acceptance criteria, including a line width at 50% peak height of the TMSP-d_4_ reference signal ≤ 1.0 Hz, and a flat baseline free of residual distortion in the water-suppression region (δ 4.5–5.0 ppm), in accordance with established high-precision ^1^H-NMR phenotyping protocols (Berton et al., 2017). The measured line width was 0.79 ± 0.10 Hz and the signal-to-noise ratio was 4180 ± 523 a.u. Water suppression was consistent across all spectra, with no distortion of neighboring resonances. All 34 spectra met these criteria and none were excluded. Analytical reproducibility was ensured by the consistent use of TMSP-d_4_ as an internal reference standard for chemical shift referencing and signal normalization, with the coefficient of variation (CV) of the TMSP-d_4_ signal remaining low across all acquired spectra (0.49 mM ± 0.001; CV = 0.3%), confirming stable spectrometer performance (Supplementary Table S1). All 48 annotated metabolites were assigned MSI level 2 (Metabolomics Standards Initiative).

### Statistical analysis

Univariate analyses comprised two complementary frameworks. Within-protocol comparisons (Before vs. After) were performed separately for the LL and HL groups using paired t-tests, and between-group comparisons (Before, After-LL, After-HL) were evaluated by one-way ANOVA. Benjamini–Hochberg false discovery rate correction was applied to both analyses across the 48 quantified metabolites, and both nominal (uncorrected) and FDR-adjusted p-values are reported. Statistical significance was set at p < 0.05.

Multivariate analyses were conducted in Python 3.12.3 using Scikit-learn (1.6.1), NumPy (2.2.0), Pandas (2.2.3), and Matplotlib (3.10.0). All metabolite values were standardized (zero mean, unit variance) prior to analysis. Principal Component Analysis (PCA) and Partial Least Squares Discriminant Analysis (PLS-DA) were applied to evaluate, respectively, the unsupervised global metabolic structure and the supervised class separation between groups.

Supervised classification was performed using the Random Forest (RF) algorithm, selected for its robustness in small-sample, high-dimensional settings, its capacity to model nonlinear relationships and interactions among correlated metabolites without assuming variable independence, and its intrinsic, Gini-based measures of feature importance, which enabled identification of the metabolites contributing most consistently to group separation across all model configurations. All models used the default Scikit-learn Random Forest Classifier configuration (100 trees, Gini impurity, max_features equal to the square root of the number of features, unrestricted depth, and bootstrap aggregation), with a fixed random_state = 42 for reproducibility. Per-model hyperparameter tuning was deliberately avoided: it would be computationally prohibitive given the exhaustive subset search described below, and aggressive tuning on a small sample would risk overfitting the cross-validation partitions; the default configuration was therefore adopted as a conservative choice, consistent with the comparatively low sensitivity of RF to hyperparameter selection.

Feature selection was not based on a single heuristic but on an exhaustive evaluation of all possible metabolite subset combinations up to the specified subset size, with the best model at each subset size selected by the highest cross-validated F1-score. Model performance was assessed by stratified 5 × 5-fold repeated cross-validation, with all reported metrics (accuracy, F1-score, and AUC) derived exclusively from out-of-fold predictions; 95% confidence intervals were estimated by bootstrap resampling (1000 iterations).

The analytical strategy followed a pre-specified hierarchical structure designed to address the three research questions in order of increasing specificity. Step 1 employed multiclass discrimination (Before, After-LL, After-HL) to assess whether the overall intervention produced distinguishable metabolomic states across all three conditions. Step 2 examined the divergence between untrained (Before) and trained (After) states, irrespective of load, as this contrast was anticipated to reflect a larger biological effect than load-specific differences. Step 3 evaluated whether HL and LL protocols produced detectably distinct profiles despite comparable hypertrophic outcomes, the more subtle and scientifically central question of the study. This sequential design reflects that the multigroup analysis in Step 1 establishes the upper bound of detectable metabolomic divergence, whereas load-specific differences require the most sensitive analytical resolution. Model robustness was confirmed by permutation testing (1000 iterations), with significance determined when the observed model AUC exceeded the 95th percentile of the permuted null distribution, following established recommendations for metabolomic machine-learning studies (Broadhurst & Kell, 2007; Westerhuis et al., 2008).

## Results

Physiological outcomes related to muscle strength, hypertrophy, and training volume following an 8-week intervention have been previously reported (Valério et al., 2023). These data are briefly summarized to provide context for the subsequent metabolomic analyses (Supplementary Table S2). The training volume per session increased substantially under both conditions. The LL group exhibited a 42% increase in leg press volume and a 56% increase in leg extension, whereas the HL group exhibited a 64% and 46% increase, respectively. These data confirm that both training modalities, despite differing in load, were similarly effective in inducing meaningful neuromuscular and morphological adaptations when performed until failure.

**Fig. 1:**
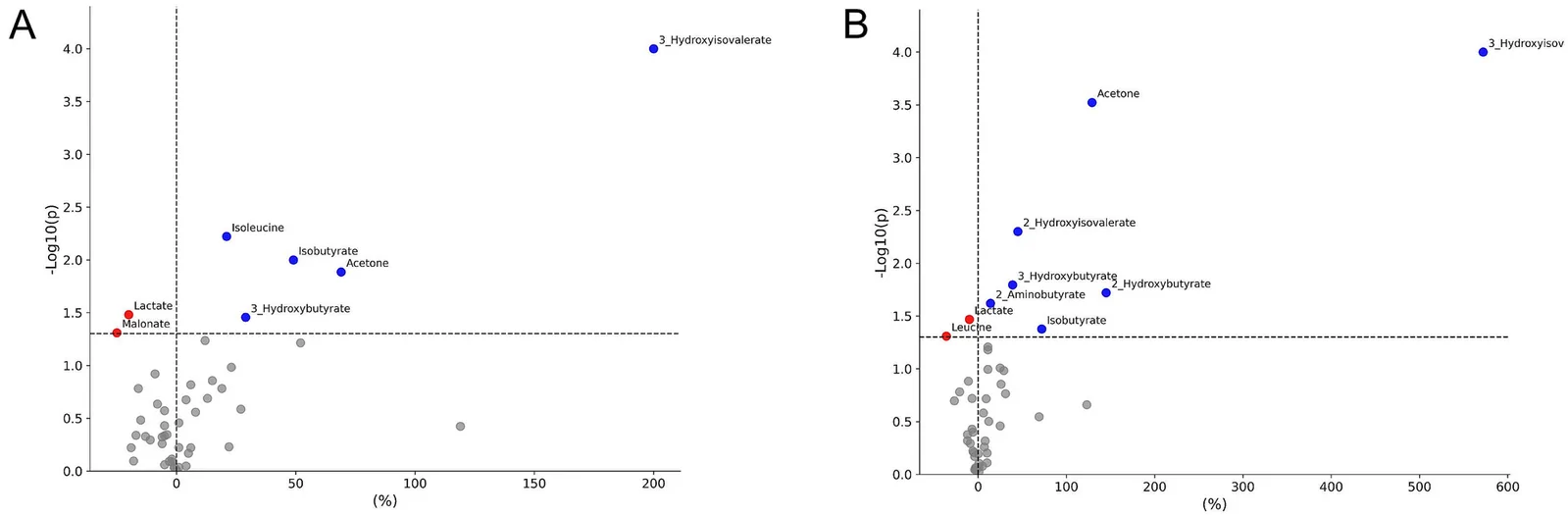
Volcano plots showing serum metabolite alterations following 8 weeks of resistance training under different load conditions: **A** low-load (LL) and **B** high-load (HL). Each point represents an individual metabolite. The x-axis displays the relative change (%), and the y-axis represents statistical significance (− log_10_(p)). Blue points indicate metabolites that were upregulated after training, while red points denote downregulated metabolites (adjusted p < 0.05). Dashed lines indicate the thresholds for statistical significance and fold-change magnitude

Figure 1 shows the metabolites that changed significantly after the 8-week resistance training intervention in the LL (Fig. 1A) and HL (Fig. 1B) groups. In total, 10 metabolites exhibited significant alterations in at least one training condition, with five metabolites (3-hydroxyisovalerate, 3-hydroxybutyrate, acetone, isobutyrate, and lactate) significantly changed in both HL and LL. This pattern suggests that both protocols share a common metabolic response to training, despite differing in mechanical load and producing comparable hypertrophic outcomes.

The untargeted NMR-based metabolomic analysis quantified 48 serum metabolites retained for analysis. Although 10 metabolites reached nominal significance (uncorrected p-value < 0.05) in at least one protocol, this signal was markedly attenuated after multiple-testing correction: following Benjamini–Hochberg adjustment, only 3-hydroxyisovalerate remained significant in both protocols, with acetone significant in the high-load protocol alone. This near-absence of individually robust metabolites underscores the limited discriminative power of single-metabolite testing and directly motivated the multivariate approach, which was subsequently applied to the complete dataset to capture the underlying covariance structure and identify complex, load-specific metabolic signatures that may not be evident through univariate comparisons alone.

Multivariate analyses were applied to the complete metabolomic dataset to evaluate global and class-specific patterns. For these analyses, all samples collected before the intervention were grouped into a single class representing the untrained condition (green, untrained), whereas the After-LL and After-HL groups corresponded to the metabolomic profiles obtained after 8 weeks of low-load or high-load resistance training, respectively (blue and red). The unsupervised PCA (Fig. S1A) revealed the overall data structure and moderate separation between the untrained and trained groups, with partial overlap between the After-LL and After-HL conditions. This indicates systemic metabolic shifts following training, particularly in the transition from untrained to trained states. The supervised PLS-DA (Fig. S1B) achieved clearer group discrimination by incorporating class membership, confirming the presence of training- and load-dependent metabolic signatures. Whereas PCA captured the main sources of variance independent of class, PLS-DA emphasized biologically meaningful separations associated with training load.

To further evaluate whether serum metabolomic profiles could effectively identify individuals according to training status, supervised models were constructed using the Random Forest algorithm. The initial three-class model (Before, After-LL, and After-HL) trained on all 48 metabolites achieved moderate overall accuracy (0.62), with imbalanced class-specific F1-scores for the LL (0.29) and HL (0.44) groups, indicating limited discriminatory power (Supplementary Table S3). 3-hydroxyisovalerate exhibited the highest feature importance in this model.

**Fig. 2:**
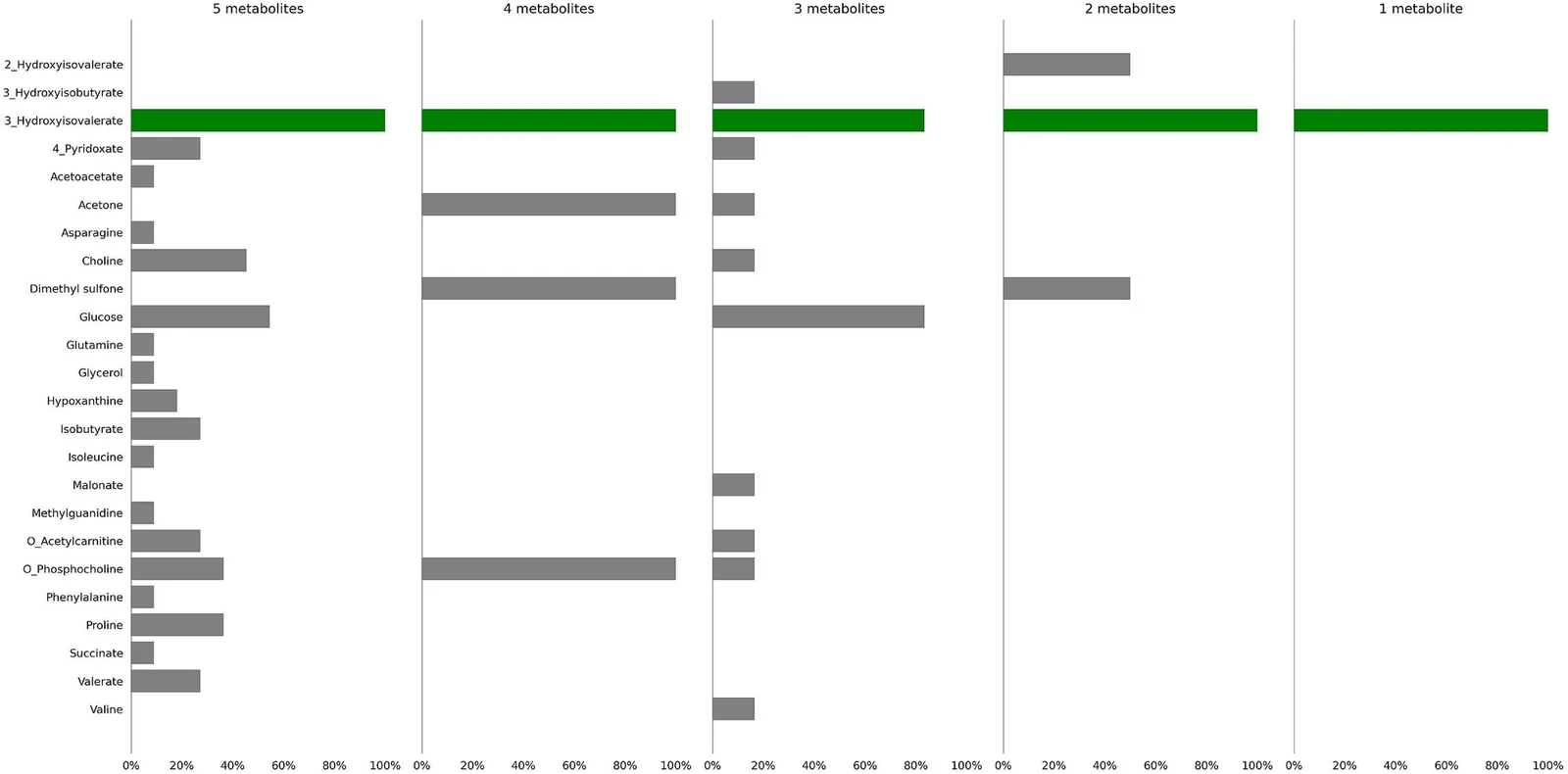
Frequency of metabolite selection among the top-performing Random Forest models used to identify the three study groups (Before, After-LL, and After-HL). Each column represents the subset size (1–5 metabolites), and bars indicate the proportion of models in which a given metabolite appeared. 3-hydroxyisovalerate (highlighted in green) was consistently selected across all subset sizes

To derive more parsimonious and robust discriminant metabolites, Random Forest models were subsequently generated using all possible combinations of one to five metabolites. Figure S1C summarizes the F1-scores of the top-performing models across subset sizes from one to five metabolites, as well as the full 48-metabolite panel. Models including three to five metabolites consistently achieved the most balanced and accurate discrimination across all groups. The best model within each subset size was selected based on the highest F1-score (Supplementary Table S3). Figure 2 presents the frequency of metabolite selection across these top models, revealing that 3-hydroxyisovalerate was the only metabolite consistently retained, highlighting its central role as a discriminator of training status.

Univariate analysis of metabolite levels was conducted using the same three-group structure as the multivariate models (Supplementary Fig. S2). Consistent with previous analyses, both training protocols (LL and HL) exhibited overlapping yet distinct biochemical response profiles, suggesting shared metabolic pathways but differing magnitudes of adaptation. Under this more conservative framework, 9 metabolites showed statistically significant changes (uncorrected p < 0.05) in at least one group, with 3 (3-hydroxyisovalerate, 3-hydroxybutyrate, and acetone) significantly altered in both the LL and HL protocols. Here too, after the Benjamini–Hochberg correction, only 3-hydroxyisovalerate and acetone remained significant, reinforcing the notion that the univariate signal is concentrated in a small number of metabolites. As expected, these three shared metabolites constitute a subset of the four identified in the within-protocol paired analysis (Fig. 1). Raw and FDR-adjusted p-values, direction of change, and fold changes for all 48 metabolites across both univariate frameworks are provided in Supplementary Table S4. Notably, the HL training protocol elicited more significant metabolic changes following the 8-week intervention, supporting the interpretation that higher training intensity amplifies systemic metabolic remodeling. It is worth noting that several metabolites found to be statistically significant in univariate comparisons were not included in the best-performing models, highlighting that univariate significance does not necessarily translate into high discriminative power in multivariate frameworks.

Considering that physiological adaptation to resistance training occurs in stages (from untrained to trained, and subsequently according to training load), a binary approach was also evaluated. In this sequential approach, the first step focused on a dual-class model comparing untrained and trained individuals (Before vs. After), regardless of load. This binary model assumes that the most pronounced metabolic distinction lies between the untrained and trained physiological states. As shown in Fig. S3A and B, the PCA and PLS-DA models revealed intrinsic group-level variance, indicating that class-specific variation accounts for a meaningful portion of the metabolomic profile. These results support the hypothesis that resistance training induces systemic, load-independent metabolic shifts detectable in serum.

Following the multivariate visualization, Random Forest models were applied to the complete metabolomic dataset to assess the degree of separation between untrained (Before) and trained (After) states. As shown in Table 1, the model incorporating all 48 metabolites achieved a cross-validated accuracy of 0.94 and balanced F1-scores for both groups (0.94 for Before and After), indicating robust and consistent group separation. However, to identify the minimum number of metabolites necessary for discriminating between untrained and trained, models using 1–3 metabolites were systematically tested.

**Table 1:** Random Forest models distinguishing between two groups (Before, After) using different metabolite subsets

Model	Accuracy	F1-score Before	F1-score After	Metabolites (feature importance)				
All 48 metabolites	0.94	0.94	0.94	Acetone (0.179)	3-Hydroxyisovalerate (0.143)	4-Pyridoxate (0.053)	Isoleucine (0.043)	Phenylalanine (0.039)
				3-Hydroxybutyrate (0.038)	Inosine (0.026)	Isobutyrate (0.022)	Glycine (0.021)	Methylsuccinate (0.021)
				O-Acetylcarnitine (0.021)	Tyrosine (0.020)	3-Hydroxyisobutyrate (0.020)	2-Hydroxyisovalerate (0.020)	Pantothenate (0.017)
				Glucose (0.016)	Alanine (0.016)	Thymidine (0.014)	Trimethylamine (0.014)	Acetoacetate (0.014)
				Asparagine (0.010)	Valerate (0.010)	2-Aminobutyrate (0.010)	Lactate (0.010)	Proline (0.010)
				Glycerol (0.010)	N-Methylhydantoin (0.010)	Creatinine (0.009)	3-Methyl-2-oxovalerate (0.008)	Leucine (0.008)
				Threonine (0.008)	Ornithine (0.007)	O-Phosphocholine (0.007)	Citrate (0.006)	Dimethyl sulfone (0.006)
				2-Oxoisocaproate (0.006)	Lysine (0.006)	Methylguanidine (0.005)	Oxypurinol (0.005)	Malonate (0.005)
				Glutamine (0.005)	2-Hydroxybutyrate (0.005)	Valine (0.005)	Methylamine (0.005)	Succinate (0.005)
				Hypoxanthine (0.004)	Pyruvate (0.004)	Choline (0.003)		
1 metabolite	0.97	0.97	0.97	3-Hydroxyisovalerate (1.000)				
2 metabolites	1.00	1.00	1.00	3-Hydroxyisovalerate (0.792)		Acetoacetate (0.207)		
	1.00	1.00	1.00	3-Hydroxyisovalerate (0.811)		Choline (0.189)		
	1.00	1.00	1.00	3-Hydroxyisovalerate (0.808)		Oxypurinol (0.205)		

Evaluation metrics and feature importance of Random Forest configurations for group separation between untrained (Before) and trained (After) states. Accuracy represents the overall proportion of correctly classified samples; the F1-score balances sensitivity and precision for each group (Before and After). Feature importance values indicate the relative contribution of each metabolite to group separation within the ensemble. All 48 metabolites were assigned MSI Level 2 confidence in accordance with the Metabolomics Standards Initiative (MSI)

Models using 3-hydroxyisovalerate alone yielded group separation comparable to the full 48-metabolite model under cross-validation, indicating that a substantial portion of the discriminative signal was concentrated in this metabolite (accuracy = 0.97; F1-score = 0.97). When combined with a second metabolite, group separation was further stabilized under cross-validation, as summarized in Fig. S3C and detailed in Table 1. These results suggest that the metabolomic divergence between untrained and trained states is largely concentrated in a small number of metabolites, with 3-hydroxyisovalerate consistently capturing the dominant portion of the discriminative signal regardless of training load. Additional metabolites, such as acetoacetate, oxypurinol, and choline, contributed meaningfully when combined with 3-hydroxyisovalerate, suggesting a context-dependent discriminative value.

To confirm that the multivariate separation between untrained and resistance-trained states was not driven by overfitting or random chance, permutation testing was performed (1000 iterations). All three top-performing models achieved AUC values of 1.00, clearly separated from the null distributions generated by label randomization (Supplementary Fig. S4). The AUC distributions from all the permuted datasets were centered below 0.80, and none of the randomized iterations reached the true model AUC. These results confirm that the observed discriminatory capacity is highly unlikely to have occurred by chance, reinforcing both the statistical robustness and biological relevance of the identified metabolite combinations.

Next, to assess whether serum metabolomic profiles differed systematically between individuals who underwent LL versus HL resistance training, a second-tier multivariate analysis was performed. As shown in Fig. S5A, PCA revealed partial overlap between the groups, whereas PLS-DA (Fig. S5B) indicated improved separation between the two training modalities, suggesting the presence of discriminative metabolic patterns. Random Forest models were applied using all 48 metabolites and systematically evaluated subsets of 1–5 metabolites (Table 2). As shown in Fig. S5C, models incorporating one to three metabolites yielded the most stable and balanced discrimination across groups, with cross-validated F1-scores exceeding 0.90 for both LL and HL conditions. In contrast, the full 48-metabolite model yielded less stable group separation, consistent with feature redundancy in a high-dimensional, small-sample setting, a finding that further motivated the systematic search for minimal informative metabolite subsets.

**Table 2:** Random Forest classification models distinguishing two groups (After-LL, After-HL) using different metabolite subsets

Model	Accuracy	F1 Classe LL	F1 Classe HL	Metabolites (feature importance)				
All 48 metabolites	0.41	0.39	0.44	Ornithine (0.053)	Methylguanidine (0.042)	Malonate (0.040)	Alanine (0.039)	Isobutyrate (0.039)
				2-Hydroxybutyrate (0.034)	2-Oxoisocaproate (0.034)	Acetoacetate (0.032)	Pyruvate (0.029)	3-Hydroxybutyrate (0.029)
				2-Hydroxyisovalerate (0.027)	Valine (0.025)	Hypoxanthine (0.033)	O-Acetylcarnitine (0.022)	Threonine (0.022)
				Glycine (0.024)	4-Pyridoxate (0.022)	Isoleucine (0.022)	Glycerol (0.022)	3-Hydroxyisobutyrate (0.021)
				O-Phosphocholine (0.036)	Creatinine (0.019)	Thymidine (0.019)	Pantothenate (0.018)	Citrate (0.015)
				Inosine (0.014)	3-Hydroxyisovalerate (0.014)	Glutamine (0.013)	Glucose (0.012)	Asparagine (0.011)
				Trimethylamine (0.011)	Leucine (0.011)	Proline (0.010)	Phenylalanine (0.009)	Methylamine (0.009)
				Choline (0.008)	Succinate (0.008)	Acetone (0.007)	Lactate (0.007)	3-Methyl-2-oxovalerate (0.007)
				Valerate (0.006)	2-Aminobutyrate (0.005)	Lysine (0.004)	Tyrosine (0.003)	Dimethyl sulfone (0.003)
				N-Methylhydantoin (0.003)	Oxypurinol (0.003)	Methylsuccinate (0.003)		
1 metabolite	0.88	0.88	0.88	O-Acetylcarnitine (1.000)				
2 metabolites	0.82	0.80	0.84	2-Oxoisocaproate (0.550)		Creatinine (0.449)		
				2-Oxoisocaproate (0.532)		Hypoxanthine (0.467)		
				Phenylalanine (0.502)		4-Pyridoxate (0.497)		
				Acetoacetate (0.547)		Choline (0.452)		
				Glucose (0.516)		Choline (0.483)		
				Malonate (0.542)		O-Phosphocholine (0.457)		
				O-Acetylcarnitine (0.910)		Valerate (0.089)		
3 metabolites	0.94	0.93	0.95	Choline (0.338)	Glucose (0.337)	Alanine (0.324)		

Evaluation metrics and feature importance of Random Forest configurations for group separation between After-LL and After-HL. AUC quantifies the overall degree of metabolomic separation between groups across all decision thresholds. Feature importance values indicate the relative contribution of each metabolite to group separation within the ensemble. All 48 metabolites were assigned MSI Level 2 confidence in accordance with the Metabolomics Standards Initiative (MSI)

**Fig. 3:**
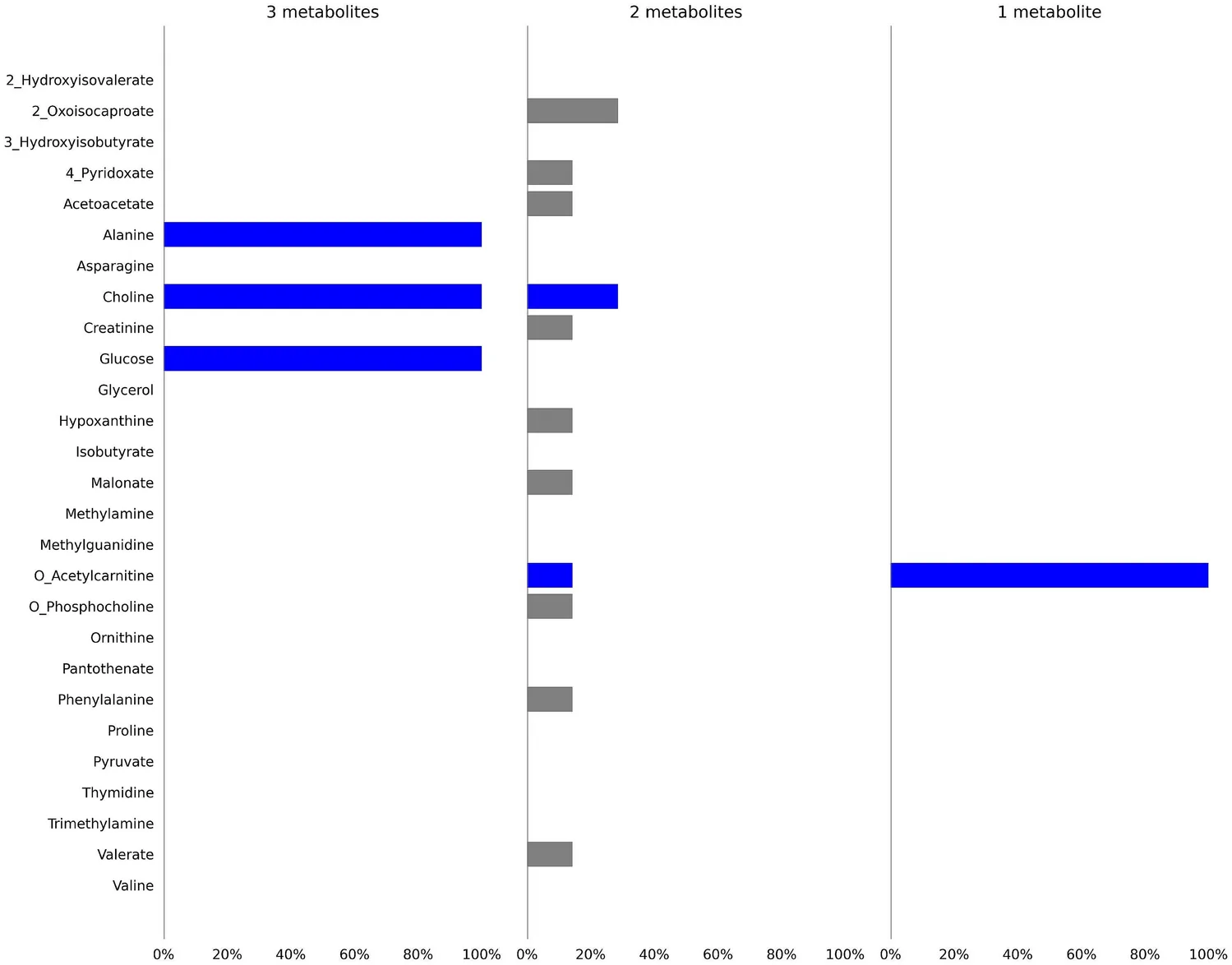
Frequency of metabolite selection across top-performing Random Forest models for discrimination of three groups (After-LL, After-HL)

Feature frequency analysis across the top-performing models (Fig. 3) revealed that metabolites such as alanine, choline, glucose, and O-acetylcarnitine were among the most frequently selected. Interestingly, unlike the binary discrimination between untrained and trained states (Table 1), where a single metabolite (3-hydroxyisovalerate) dominated, this step showed a broader distribution of relevant features, reflecting subtle metabolic differences between individuals after performing the LL and HL protocols. As only one combination of three metabolites yielded stable group separation between LL and HL states: choline, glucose and alanine; permutation testing was conducted to confirm robustness (Supplementary Fig. S6). The configuration achieved an AUC of 0.94, significantly exceeding the permuted null distribution across all 1000 iterations, confirming that the observed metabolomic divergence was unlikely to have arisen by chance. These results suggest that, although the metabolomic divergence between LL and HL is more subtle than that between untrained and trained states, a distinct and reproducible biochemical signal is nonetheless detectable through the joint modelling of specific metabolite combinations.

**Fig. 4:**
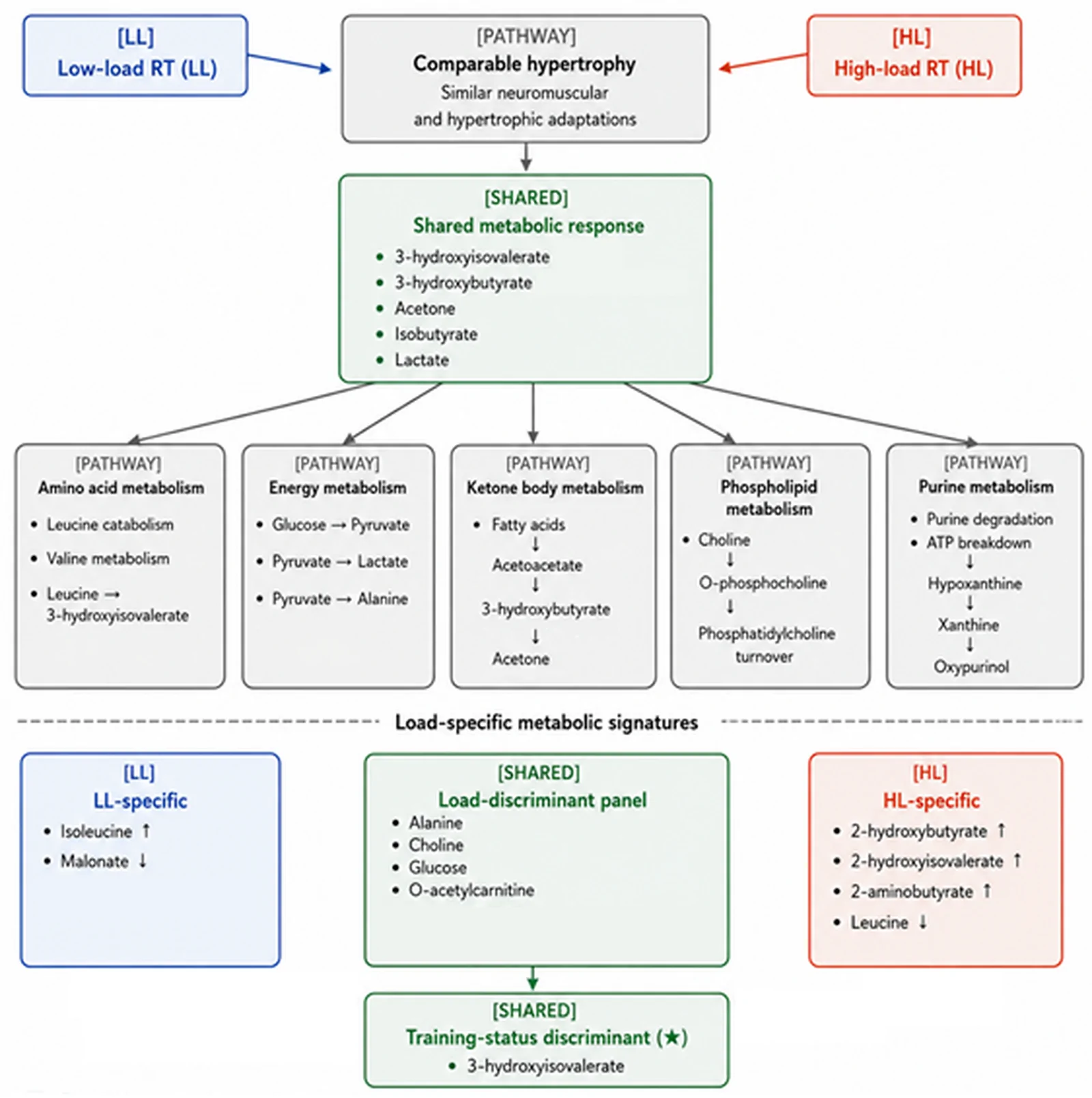
Metabolic pathway integration of resistance training adaptations. Discriminant metabolites were manually mapped onto established metabolic pathways using KEGG as the primary reference database, based on their known biochemical identities and published roles in exercise metabolism. The shared metabolomic response (central panel) encompasses metabolites consistently altered across both HL and LL protocols, anchored by 3-hydroxyisovalerate (★) as the primary training-status metabolite arising from leucine catabolism. LL-specific metabolic alterations (blue); HL-specific metabolic alterations (orange); Shared metabolic response LL and HL (green); and biochemical pathways and structural components (Grey)

To contextualize the multivariate findings within established exercise biochemistry, the consistently selected discriminant metabolites were mapped onto known metabolic pathway frameworks (Fig. 4). The shared metabolomic response to RT, anchored by 3-hydroxyisovalerate, 3-hydroxybutyrate, acetone, isobutyrate, and lactate—mapped coherently onto three interconnected pathway clusters: leucine and valine catabolism, ketone body turnover, and glycolytic–oxidative rebalancing.

## Discussion

This study provides new insights into how human metabolism responds to resistance training (RT), with a specific focus on identifying metabolite combinations that can distinguish between different training statuses and loads in healthy young men. The main findings can be summarized as follows: (1) Serum metabolic signatures successfully differentiated untrained from trained individuals, reflecting systemic adaptations to RT. 3-hydroxyisovalerate emerged as the most consistently discriminant metabolite distinguishing trained from untrained participants; (2) Compact metabolite combinations (3-hydroxyisovalerate paired with acetoacetate, choline, or oxypurinol) proved informative for characterizing the metabolomic shifts induced by RT; (3) Low- and high-load training protocols resulted in distinct metabolic signatures, with HL exercise producing more pronounced systemic metabolic perturbations. A metabolite combination comprising choline, glucose, and alanine emerged as a hypothesis-generating signature for characterizing load-specific metabolomic divergence, warranting prospective investigation in larger, independent cohorts. Five metabolites (3-hydroxybutyrate, acetone, isobutyrate, 3-hydroxyisovalerate, and lactate) showed consistent changes across training loads, suggesting that both resistance training protocols induce shared systemic metabolic adaptations. These changes suggest a broader reorganization of systemic substrate utilization, including shifts in ketone body metabolism, lipid oxidation, glucose utilization and handling, as well as amino acid turnover induced by chronic RT (Berton et al., 2017).

The elevation of 3-hydroxybutyrate and acetone is consistent with altered ketone body turnover, potentially suggesting adaptations in lipid-derived energy utilization, as described in the exercise physiology literature (Ehtiati et al., 2025; Pali et al., 2025). Although 3-hydroxybutyrate has been characterized in other contexts as a signaling metabolite capable of modulating transcriptional programs (Koutnik et al., 2019; Møller, 2020; Soni et al., 2024), the present findings more conservatively indicate modifications in circulating substrate availability. Similarly, elevations in acetone, derived from acetoacetate metabolism, are compatible with increased ketone body turnover, which has been associated with enhanced oxidative metabolism during sustained training adaptation. Furthermore, the reduction in circulating lactate concentrations may reflect altered lactate production–clearance dynamics or adjustments in the systemic oxidative–glycolytic balance (Evans et al., 2017). Isobutyrate and 3-hydroxyisovalerate are linked to branched-chain amino acid (BCAA) metabolism. Their coordinated modulation suggests alterations in amino acid turnover and possible redistribution across tissues in response to repeated contractile stimulus demand (Beaumont & Blachier, 2020).

In multivariate analysis, these metabolomic differences were consistently associated with group separation between trained and untrained individuals, particularly when 3-hydroxyisovalerate was included alone or in combination with minimal panels of up to 5 metabolites (Fig. 2). 3-hydroxyisovalerate is a downstream product of leucine degradation, and its elevation suggests modulation of leucine-derived metabolic pathways following chronic RT (Wilkinson et al., 2013). Leucine plays a well-established role in anabolic signaling and skeletal muscle protein metabolism (Karlsson et al., 2004; Neishabouri et al., 2015). Thus, the increase in leucine-derived metabolites observed here is consistent with altered leucine turnover and a possible redistribution of substrate between oxidation and incorporation into protein pools during muscle remodeling. This association, however, remains hypothesis-generating and would require targeted approaches such as isotope tracing for mechanistic confirmation. The near-perfect separation between untrained and trained states (AUC = 1.00) should be interpreted in light of the sample size. Although permutation testing rendered a chance result highly unlikely, such a value in a 17-participant cohort most plausibly reflects a large, biologically expected training effect amplified by low sample dimensionality. It should therefore be read as evidence that a robust training-status signal exists, not as accuracy expected in an independent cohort. Moreover, although the paired Before-versus-After design increases sensitivity by controlling for stable between-subject variation, it does not fully exclude time-related or other non-training-related contributors to the observed metabolomic shift. In the absence of a non-exercising control arm over the same period, the near-perfect discrimination should be read as consistent with, rather than as isolated proof of, a training-specific effect.

A critical consideration is that 3-hydroxyisovalerate may arise through mechanisms other than exercise-induced leucine catabolism. Two alternative explanations warrant explicit acknowledgment. First, 3-hydroxyisovalerate may be detectable in contexts involving leucine-derived supplementation, as exogenous leucine loading increases substrate availability for the 3-MCC pathway independently of training-induced turnover. Second, and mechanistically distinct, marginal biotin insufficiency may impair 3-MCC activity given its obligate dependence on biotin as a cofactor, diverting 3-methylcrotonyl-CoA flux toward the 3-hydroxyisovalerate shunt regardless of leucine catabolic rate (Mock et al., 2011). Experimental biotin depletion studies have demonstrated that urinary 3-hydroxyisovalerate excretion increases significantly within the first days of subclinical insufficiency, often preceding other biochemical indicators of compromised biotin status (Stratton et al., 2011). Several factors, however, support an endogenous adaptive interpretation in the present study. First, the longitudinal within-subject design reduces the likelihood that inter-individual dietary variability accounts for the observed changes. Second, the increase in 3-hydroxyisovalerate occurred in parallel with coordinated alterations in other metabolites involved in BCAA and energy metabolism, suggesting integrated metabolic remodeling rather than isolated exposure to a single compound. Third, blood sampling was conducted under fasting conditions with standardized dietary guidance before collection. Taken together, these considerations suggest that the observed 3-hydroxyisovalerate pattern is more consistent with training-associated modulation of amino acid turnover in presumably biotin-replete young adults than with acute supplementation effects, although the relative contribution of subclinical biotin insufficiency cannot be excluded without direct assessment of biotin status markers.

A key finding of this study was the consistent selection of 3-hydroxyisovalerate across top-performing models when associated with other metabolites, such as acetoacetate, oxypurinol, or choline. These metabolite combinations suggest that RT induces biochemical shifts that extend across broader intermediary metabolism (Fig. 4). Choline emerged as a relevant feature across several multivariate configurations. This metabolite is involved in phospholipid metabolism and acetylcholine biosynthesis, thereby linking membrane structural dynamics to neuromuscular function (Maia et al., 2025). In skeletal muscle, choline serves as a precursor for membrane phospholipids, particularly phosphatidylcholine, and contributes to processes related to excitation–contraction coupling (Moretti et al., 2020). Adequate phosphatidylcholine availability is essential for maintaining mitochondrial integrity and metabolic homeostasis, modulating inflammatory signaling pathways, and contributing to the regulation of muscle pain and inflammatory responses (Kusuda et al., 2020; van der Veen et al., 2017). In parallel, oxypurinol, which is derived from xanthine oxidase inhibition and has been associated with reduced oxidative stress in clinical settings (Ryan et al., 2011; Sanchis‐Gomar et al., 2015), was retained in specific multivariate configurations. Importantly, within multivariate frameworks such as RF, variable importance should not be interpreted as reflecting hierarchy; rather, it represents the relative statistical contribution of each metabolite to group separation within an interdependent metabolic network. Thus, the recurrent selection of these metabolites likely reflects coordinated metabolic patterning characteristic of the trained phenotype.

Among the recurring metabolites, O-acetylcarnitine was associated with metabolomics divergence between sedentary and trained states and also contributed to group separation between HL and LL training protocols (Table 2). These findings are consistent with those of Hayden et al. (2024), who observed an association between the O-acetylcarnitine content in skeletal muscle and the capacity to generate ATP. They found that this capacity was reduced when there was a greater reliance on non-oxidative glycolysis and a lower contribution from oxidative phosphorylation. This characteristic of O-acetylcarnitine as an adaptive molecule in response to metabolic demands suggests it may be relevant for characterizing both untrained individuals, who have a limited capacity to oxidize fatty acids as an energy substrate, and trained subjects, in whom oxidation is more efficient. Consistent with the above, Castro et al. (2024) found that O-acetylcarnitine is a urinary marker of cardiorespiratory fitness and cardiorespiratory function (Castro et al., 2024). The present findings further the relevance of this metabolite in characterizing training status and metabolic adaptation. However, it should be stressed that no single metabolite is sufficient owing to the large inter-individual variability in metabolic responses, as extensively discussed previously (Kelly et al., 2020). This highlights the importance of limited but multi-marker panels that offer a broader perspective.

Resistance-training adaptation has only recently begun to be characterized at the molecular level, within what has been framed as an emerging domain of resistomics aimed at mapping the multi-omic signatures of hypertrophic and neuromuscular adaptation in accessible biofluids (Khoramipour et al., 2025). Most exercise metabolomics, however, has characterized the immediate response to a single bout (Pellegrino et al., 2022), and the chronic signature is a distinct entity, since only a fraction of acutely altered metabolites persists after training (Gehlert et al., 2022). Chronic resistance training does reshape the resting metabolome, as Sarin et al. (2019) showed through antiatherogenic serum shifts after sixteen weeks of training. Yet the comparison of training loads has remained largely unexplored. The one acute comparison from our group found the load difference real but metabolite-sparse, with high-load exercise raising pyruvate, lactate, and alanine relative to low-load training under blood-flow restriction, and choline diverging in the low-load condition (Valério et al., 2018). Notably, alanine and choline reappear in the present chronic signature, no longer as isolated shifts but as covarying components of the load-discriminative panel. That metabolomics can resolve exercise-mode-specific signatures is by now well established (Morville et al., 2020), and even the comparison of resistance-training types yields only modest separation in well-powered serum cohorts (Feuerbacher et al., 2025); whether it could resolve the subtler contrast between loads of a single mode remained the open question. The present findings provide initial evidence that training load leaves a coordinated imprint on the circulating metabolome that persists even when high- and low-load protocols converge to achieve equivalent hypertrophy. Consistent with this, the degree of group separation observed here, which varied from single-metabolite configurations to those combining up to five metabolites, revealed a consistent pattern: separation of untrained from trained states was robust across different multivariate configurations (Table 1). Detecting metabolomics divergence between trained groups using LL and HL protocols proved more subtle, likely due to overlapping metabolic responses. Despite this, the distinct metabolic profiles observed between the HL and LL training groups support the interpretation that training load meaningfully influences systemic metabolism.

The observed elevations in 2-hydroxybutyrate, 2-hydroxyisovalerate, and 2-aminobutyrate alongside reduced leucine concentrations in the HL group are consistent with increased branched-chain amino acid (BCAA) catabolism and perturbation of amino acid metabolic equilibrium during intense exercise (Wagenmakers, 1998). The accumulation of these metabolites, together with decreased leucine, supports the interpretation that high-load training is associated with greater systemic perturbation of amino acid metabolic equilibrium. This is further corroborated by studies showing that exercise increases BCAA turnover and alters the balance of amino acid pools, with downstream effects on energy metabolism and nitrogen balance (Glynn et al., 2015; Neinast et al., 2019). In contrast, the LL group showed decreased malonate levels, accompanied by increased isoleucine, suggesting a more selective metabolic response of lower overall magnitude than that observed under HL (Fig. 1). Analysis of the frequency of metabolite selection across the top-performing RF configurations (Fig. 3) revealed that the metabolites most frequently contributing to group separation between the HL and LL groups were alanine, glucose, and choline. Glucose functions as the primary rapidly mobilizable substrate for ATP production during high-intensity muscle work (Pi et al., 2023), particularly under conditions approaching muscular failure. Under such metabolic stress, alanine may contribute to energy homeostasis through amino acid catabolism, anaplerotic support of the TCA cycle, and participation in gluconeogenesis (Lewis et al., 2010; Pi et al., 2023). Importantly, when assessed individually, none of these metabolites reached univariate significance between the post-training states, showing only minor and non-significant directional changes (choline: − 11% HL, + 3% LL, p > 0.7; glucose: − 9% LL, − 1% HL, p > 0.4; alanine: + 2% in both, p > 0.9). Their contribution to load discrimination therefore cannot be explicated to the magnitude or direction of change in any single metabolite. Rather, the relevance of glucose, alanine, and choline lies in their joint selection and coordinated covariance among them, spanning carbohydrate availability, amino acid–carbohydrate coupling, and membrane phospholipid turnover—that yields reproducible load-discriminative information when the metabolites are analyzed together. This reinforces the central rationale of the study: the load-dependent signal does not reside in any isolated metabolite but in the joint structure linking them, a structure recoverable only through multivariate modelling (Broadhurst & Kell, 2007).

This study has several limitations that must be considered when interpreting its findings. (1) The relatively homogeneous demographic characteristics of the sample (healthy young men of similar age and training background) may have reduced biological variability and contributed to the degree of group separation observed. Future studies including women, broader age ranges, and heterogeneous training backgrounds are necessary to assess the generalizability of these findings. (2) The small final sample size (n = 17) inherently restricts statistical power and increases the risk of overfitting. Although stratified 5 × 5-fold repeated cross-validation, permutation testing (1000 iterations), and bootstrap confidence intervals were applied, the possibility of optimistic bias cannot be completely excluded. Perfect or near-perfect group separation in some configurations should be interpreted cautiously as cohort-specific patterns rather than fully generalizable biological signatures, consistent with previous recommendations (Broadhurst & Kell, 2007; Westerhuis et al., 2008). (3) The study lacks a non-exercising sedentary control group across the same 8-week observation window. Without a comparator, the contribution of temporal metabolic drift or unmeasured lifestyle confounders (particularly to the near-perfect Before-versus-After discrimination) cannot be fully excluded; future randomized controlled trials should incorporate a control arm. (4) The exclusive use of 1H-NMR provides lower sensitivity for low-abundance signaling metabolites compared with mass spectrometry-based platforms; complementary LC–MS/MS profiling in future work would yield a more complete metabolic picture. (5) Although samples were collected under standardized 10-h fasting conditions with dietary recording, systematic exclusion of leucine-derived supplementation by controlled feeding or isotope tracing was not implemented; future studies should apply targeted isotope tracing to distinguish endogenous leucine catabolism from exogenous supplementation. (6) Total training volume (sets × repetitions × load) and time-to-concentric-fatigue were not equated between protocols; differences in total mechanical work may have contributed to the observed metabolic divergence. (7) Biotin status was not assessed, and marginal biotin insufficiency cannot be excluded as an alternative mechanism for the observed 3-hydroxyisovalerate elevations. (8) Finally, dedicated pooled QC samples were not acquired. In summary, this study demonstrates that resistance training induces coherent and biologically interpretable metabolic shifts, with 3-hydroxyisovalerate consistently emerging as a central marker of training status. The biological pathway integration framework contextualizes these findings within established exercise biochemistry, supporting the biological coherence of the multivariate-derived signatures (Fig. 4). These results should be regarded as hypothesis-generating and require replication in larger, independent cohorts before applied or clinical implementation.

## Conclusion

The present study demonstrates that a multivariate analytical framework can reveal load-specific metabolic signatures of resistance training that remain inaccessible to conventional univariate approaches. Although univariate testing identified a subset of metabolites altered by training, it was only through the joint modelling of the full metabolomic profile that the more subtle distinction between HL and LL conditions became apparent. This finding has direct physiological relevance: the metabolic response to resistance training is not adequately captured by individual metabolite changes in isolation but emerges from coordinated shifts across interconnected pathways involving amino acid turnover, substrate utilization, and systemic energy remodeling. Machine learning models, applied here in an exploratory and hypothesis-generating capacity, served as a tool to operationalize this multivariate perspective—not to establish predictive panels for applied use, but to interrogate which combinations of metabolites carry discriminative physiological information. The biological pathway integration framework (Fig. 4) contextualizes the machine-learning-derived signatures within established exercise biochemistry, mapping the discriminant metabolites onto their respective metabolic pathways and providing biological plausibility for the identified metabolomic signatures. These findings should be regarded as preliminary, given the small sample size and the absence of external validation. Nevertheless, they provide a conceptual and biological foundation for future studies to examine whether multivariate metabolomic analyses can contribute to a more complete and biologically nuanced characterization of the adaptive response to resistance training.

## Supplementary Information

Below is the link to the electronic supplementary material.

Supplementary file1 (DOCX 825 kb)

Supplementary file2 (XLSX 9 kb)

## Data Availability

The metabolomics data reported in this paper are available via MetaboLights https://www.ebi.ac.uk/metabolights/MTBLS15091 study identifier MTBLS15091. The data analysis code is publicly available on GitHub and permanently archived in Zenodo (https://doi.org/10.5281/zenodo.20564410).
