# Supplementary material for: Serum metabolomic profiling reveals load-specific adaptations to resistance training
Source: Metabolomics. 2026 Aug 23;22(5):144. doi: 10.1007/s11306-026-02514-5 (PMC13500427; doi:10.1007/s11306-026-02514-5)
Supplement: Supplementary file 1 [file 11306_2026_2514_MOESM1_ESM.docx]

**Supplementary Material**

Serum Metabolomic Profiling Reveals Load-Specific Adaptations to Resistance Training

Diego Salgueiro^1^ [^0000-0002-5712-1546^](https://orcid.org/0000-0002-5712-1546) · Matthews Martins^2, 5^ [^0000-0002-0050-4490^](https://orcid.org/0000-0002-0050-4490) · Guilherme Scherrer^3^ [^0009-0009-0030-1042^](https://orcid.org/0009-0009-0030-1042) · Denis Valério^4^ [^0009-0001-5910-6307^](https://orcid.org/0009-0001-5910-6307) · Alex Castro^6^ [^0000-0002-0298-2672^](https://orcid.org/0000-0002-0298-2672) · Renato Barroso^4^ [^0000-0001-8112-6622^](https://orcid.org/0000-0001-8112-6622) · Richard Leite^3^ [^0000-0001-7937-6972^](https://orcid.org/0000-0001-7937-6972) · Valerio Barauna^2, 5,^* [^0000-0003-2832-0922^](https://orcid.org/0000-0003-2832-0922)

^1^School of Physical Education and Sport of Ribeirão Preto, University of São Paulo, Ribeirão Preto, Brazil

^2^Department of Physiological Sciences, Federal University of Espírito Santo, Vitória, Brazil

^3^Laboratory of Strength and Conditioning, LAFEC, Center for Physical Education and Sports, Federal University of Espírito Santo, Vitória, Brazil.

^4^Physical Education Faculty, Sports Science Department, State University of Campinas.

^5^National Institute of Science and Technology - INCT Physical (In)activity and Health, CNPq, Brazil

^6^Brazilian Biosciences National Laboratory (LNBio), Brazilian Center for Research in Energy and Materials (CNPEM), São Paulo, Brazil

***** Correspondence: [valerio.barauna@ufes.br](mailto:valerio.barauna@ufes.br); Tel.: +55 27 996892407

2 Methods
  2.1.1 Detailed description of Screening

As a result of screening during the interview, 30 healthy young male volunteers were eligible to participate in the study, all of whom had not participated in resistance training programs in the previous 12 months. Individuals with metabolic diseases, such as diabetes, were excluded from the study, as were those on caloric-restricted diets and those with bone, muscle, or joint issues that could affect their physical performance. Of the initial 30 participants, five dropped out for personal reasons, leaving 25 participants who were randomly assigned to the study conditions. In the HL group, four participants were excluded: one due to a participation rate of < 90% and three due to two or more consecutive absences. In the LL group, four participants were excluded: two for a participation rate < 90% and two for missing more than two consecutive sessions. Therefore, participants who failed to attend at least 90% of the training sessions or missed more than two consecutive sessions were excluded from the study. Consequently, 17 participants (nine in the HL group and eight in the LL group) were included in the final analysis.

2.2.1 Detailed Description of the 8-Week Resistance Training Program

The participants completed two training sessions to familiarize themselves with the 1-RM test for the 45° leg press and bilateral leg extension exercises. Following these sessions, a 1-RM test and retest were conducted. The minimum interval between the laboratory visits was 72 h. After the retest, an eight-week training program was implemented. Participants performed the 1-RM test after the fourth week of training and 72 h before the final training session to adjust their loads. This procedure ensured that training was conducted at the correct intensity, particularly during the final session, when metabolic responses were analyzed.

Maximum strength was measured using the 1-RM test, following the guidelines of the American College of Sports Medicine for leg press and leg extension exercises (Brown L.E. 2001). Before the test, the participants performed a 5-minute warm-up on a stationary bike. This was followed by a specific warm-up consisting of two sets with eight and three repetitions, respectively, using estimated loads of 50% and 70% of the 1-RM weight. There was a 3-minute interval between the end of the warm-up and the start of the test. All the participants performed the tests in the same order. The initial weight was estimated during the familiarization sessions and gradually increased until the participant could not complete the repetitions. The total number of attempts to determine the 1-RM did not exceed five. A 3-minute interval was allowed between each attempt and 10 min between exercises.

Participants were instructed to maintain the same dietary habits as on the day before blood collection. Experienced researchers guided participants in completing the dietary record for the previous day. Total energy intake and the proportion of macronutrients (carbohydrates, lipids, and proteins) were analyzed using NutWin software (v. 1.5, University of São Paulo). After a 10-hour fast, blood samples were collected from the arm vein using serum tubes (Vacuette, 8 mL). The participants also consumed a standardized meal (290 kcal, 60% carbohydrates, 25% lipids, and 15% proteins) one hour before each blood collection timepoint. All protocols were performed between 8 a.m. and 11 a.m. to avoid circadian rhythm influence.

2.3.1 Detailed description of blood metabolome via ^1^H-NMR

Venous puncture was performed via the median cubital vein located in the antecubital fossa. After collection (8 mL), blood was allowed to rest for 30 minutes at room temperature. Following this period, samples were centrifuged for 10 minutes at 3,000 RPM at room temperature. After this step, approximately 4 mL of serum was stored at −80°C. Before analysis, a 3 kDa filter (Amicon Ultra, Sigma-Aldrich, USA) was washed using 500 µL of Milli-Q water. After applying Milli-Q water to the filter, it was centrifuged at 14,000 RPM for 10 minutes at 4°C. This process was repeated five times. Following this washing step, 350 µL of previously stored serum was loaded onto the filter and centrifuged at 14,000 RPM for 45 minutes at 4°C. The filtrate (200 µL) was then recovered and transferred to a 5 mm NMR tube (Wilmad, Sigma-Aldrich, USA) (Shimizu et al. 1994).

Spectral acquisition was performed by ^1^H-NMR spectrometer at 600MHz equipped with a triple-resonance cryogenic inverse probe (Varian, Agilent Inova). The sample tuning adjustments with the device were performed by a specialized technician, and the other adjustments (lock and shimming) were performed manually for each sample. The analysis was performed at a constant temperature of 298 K (25 °C). The spectrum was acquired via 256 scans, with an interval of 1.5 seconds and an acquisition time of 4 seconds for each scan. The processing of the ^1^H-NMR spectral data was performed using the Chenomx® NMR Suite 7.6 software (Chenomx Inc., Edmonton, AB, Canada). Spectral processing consisted of spectral phase adjustment, baseline correction, and signal removal from water (4.6–5.1 ppm). Then, a 0.3 Hz line-broadening apodization function was applied, and the spectrum was calibrated by the internal reference signal (TMSP-d4). The Chenomx® NMR Suite 7.6 software (Chenomx Inc.), along with the Human Metabolome Database - HMDB 4.0 (Wishart et al. 2018), was used to identify the metabolites. Quantitative analyses were performed with the integral of signals of each metabolite.

Spectral quality was evaluated against a set of predefined acceptance criteria established for high-precision ¹H-NMR phenotyping, comprising a line width at 50% peak height of the TMSP-d₄ reference signal not exceeding 1.0 Hz, and a flat, distortion-free baseline throughout the water-suppression region (δ 4.5–5.0 ppm). The observed line width of 0.79 ± 0.10 Hz and a signal-to-noise ratio of 4,180 ± 523 a.u. confirmed that instrumental performance was well within the defined thresholds. Water suppression was uniform and reproducible across all spectra, with no detectable distortion of adjacent resonances. All 34 spectra fulfilled the established quality criteria; consequently, no spectra were excluded from subsequent analyses. Analytical reproducibility was further substantiated by the systematic use of TSP-d₄ as an internal reference standard for both chemical shift calibration and signal normalization. The coefficient of variation (CV) of the TSP-d₄ signal was consistently low across the entire dataset (0.49 mM ± 0.001; CV = 0.3%), providing robust evidence of spectrometer stability throughout the analytical run (Supplementary Table S1). All 48 annotated metabolites were assigned confidence level 2 according to the Metabolomics Standards Initiative (MSI) reporting framework.

2.4.1 Detailed Description of Statistical Analysis

For within-group comparisons (Before vs. After) in LL and HL, paired t-tests were performed for each metabolite. Between-group comparisons (Before, After-LL, and After-HL) were evaluated using one-way ANOVA. Metabolomic data are expressed as fold changes relative to the Before condition. A significance threshold of p < 0.05 was adopted. To account for multiple comparisons when testing the full panel of 48 metabolites, p-values from the paired t-tests were adjusted using the Benjamini–Hochberg false discovery rate (FDR) correction. Multivariate analyses were conducted using the Scientific Python Development Environment (Spyder 6.0.3) and Python version 3.12.3. The libraries employed included Scikit-learn (1.6.1), NumPy (2.2.0), Pandas (2.2.3), and Matplotlib (3.10.0).

Random Forest was selected due to its robustness in small-sample, high-dimensional settings, its ability to model nonlinear relationships, and its resistance to overfitting through bootstrap aggregation. Additionally, RF provides intrinsic measures of feature importance, which enabled the identification of the metabolites contributing most consistently to group separation across all model configurations. Simpler linear models were not prioritized because preliminary exploratory analyses indicated potential nonlinear interactions among metabolites. RF is a supervised ensemble method that combines multiple decision trees to produce a robust aggregate output, offering greater stability than any individual tree while capturing nonlinear covariance structures inherent to metabolomic data. Each tree was built independently using bootstrap sampling, with a random subset of metabolites evaluated at each node. The final output was determined by majority vote across all trees. Additionally, RF provides intrinsic feature importance scores, which quantify the relative contribution of each metabolite to group separation across the ensemble — enabling identification of the metabolites most consistently associated with the observed metabolomic divergence between groups (Breiman 2001). Model validation employed a stratified K-Fold Cross-Validation strategy (k=5) repeated five times (5×5-fold repeated CV) (Pérez-Guaita et al. 2015). In this procedure, all samples are used for both training and testing, such that each sample is included in the held-out set exactly once. Final performance metrics were computed from the aggregated out-of-fold results across all individuals, ensuring that reported values reflect group separation on unseen data. The evaluation metrics included accuracy, which represents the overall proportion of correctly assigned samples, and the F1-score, which balances sensitivity and precision for each group. The F1-score was selected as the primary evaluation metric because it provides a robust measure of group separability by combining sensitivity and precision into a single interpretable value (Hinojosa Lee et al. 2024). The models were constructed using a full set of 48 metabolites and reduced subsets of variables (metabolites). Specifically, 48 models were constructed using one metabolite, 1,275 using two, 20,825 using three, 249,900 using four, and over two million using five or more metabolites. To identify the minimal metabolite subset sufficient to capture the metabolomic divergence between groups, an exhaustive search algorithm systematically evaluated all possible combinations of one to five metabolites, proceeding until evaluation metrics stabilized or reached a plateau — indicating that additional metabolites contributed negligible incremental information to group separation.

Given the hierarchical structure of the research question — first characterizing the metabolomic divergence between untrained and trained physiological states, and subsequently examining whether load-specific biochemical differences were detectable among trained individuals — a sequential analytical framework was pre-specified. Accordingly, three analytical tasks were conducted: (1) a multigroup model examining separation among Before, After-LL, and After-HL states; (2) a binary model assessing the metabolomic divergence between untrained (Before) and trained individuals (After, irrespective of load); and (3) a binary model evaluating load-specific metabolomic differences between After-LL and After-HL. This sequential structure enabled assessment of the dominant training-induced metabolomic shift before interrogating the more subtle load-dependent biochemical divergence.

Permutation testing was performed to confirm that the observed group separation was not attributable to overfitting or random chance (Broadhurst and Kell 2007). This approach involves randomizing the class labels to generate a null distribution of evaluation metrics under conditions where no true metabolomic divergence exists between groups. For both binary analytical tasks — untrained vs. trained and After-LL vs. After-HL — 1,000 permuted models were generated by randomly shuffling class assignments across all iterations. For each permuted model, the AUC was computed and compiled into a null distribution. The observed model AUC was considered statistically significant when it exceeded the 95th percentile of the permuted distribution (p < 0.05), confirming that the degree of group separation was unlikely to have arisen by chance. The model submitted to permutation testing was built using the smallest metabolite subset at which evaluation metrics plateaued under stratified 5×5-fold repeated cross-validation, thereby minimizing the risk of overfitting while identifying the metabolites most consistently associated with the observed metabolomic divergence.

To visualize discrimination of samples based on interactions among all 48 metabolites, two methods were applied: Principal Component Analysis (PCA), an unsupervised approach, and Partial Least Squares Discriminant Analysis (PLS-DA), a supervised approach. PCA is a multivariate technique that focuses on dimensionality reduction and can extract information from a set of intercorrelated variables, representing them in a new space defined by orthogonal variables, known as principal components. It is considered an unsupervised method because it does not rely on predefined labels or classes. Instead, it identifies natural patterns and clusters inherent in the data, which are visually represented in a score plot. PLS-DA is a supervised technique that requires prior knowledge of each sample's class membership. Accordingly, PLS-DA performs dimensionality reduction while incorporating class label information and maximizes the covariance between the predictors and response variables. The resulting latent variables, which can be visualized in a score plot, were specifically constructed to optimize class separation, thereby improving discrimination and classification performance. Before analysis, the data were standardized using StandardScaler and labeled by group. Subsequently, figures were generated with confidence ellipses representing each class, corresponding to a 95% confidence interval.

**Supplementary Tables**

**Table S1** – Analytical quality-control metrics for the ¹H-NMR acquisition sequence.

| **Acquisition quality-control parameter** | **Value (across 34 acquisitions)** |
| --- | --- |
| TMSP-d_4_ reference-signal position (δ) | 0.00 ± 0.001 ppm |
| mM (TMSP-d_4_) | 0.49 ± 0.001 Hz |
| %CV (TMSP-d_4_) | 0.3% |
| Signal-to-noise ratio (TMSP-d_4_) | 4.180 ± 523 a.u. |
| Spectra excluded | 0 of 34 |

_Values represent mean ± standard deviation across 34 acquisitions. Chemical shift calibration was performed using TMSP-d4 as internal reference (δ 0.00 ppm). ¹H-NMR: proton nuclear magnetic resonance; TMSP-d4: sodium 3-(trimethylsilyl)-2,2,3,3-tetrapropionate-d4; δ: chemical shift; %CV: coefficient of variation; SNR: signal-to-noise ratio; a.u.: arbitrary units._

**Table S2** - Summary of physiological adaptations in strength, hypertrophy, and training volume after 8 weeks of resistance training.

|  |  | **LL** | | |  |  |  | **HL** | | |  |  |
| --- | --- | --- | --- | --- | --- | --- | --- | --- | --- | --- | --- | --- |
|  |  | **Before** |  | **After** |  | **p** |  | **Before** |  | **After** |  | **p** |
| **Strength** |  |  |  |  |  |  |  |  |  |  |  |  |
| 1RM Leg Press (Kg) |  | 245.6 ± 44.8 |  | 282.5 ± 59.2 |  | **<0.05** |  | 270.6 ± 82.6 |  | 321.1 ± 96.0 |  | **<0.05** |
| 1RM Leg Extension (Kg) |  | 89.8 ± 23.0 |  | 101.4 ± 17.0 |  | **<0.05** |  | 86.8 ± 20.7 |  | 102.9 ± 19.6 |  | **<0.05** |
|  |  |  |  |  |  |  |  |  |  |  |  |  |
| **Hypertrophy** |  |  |  |  |  |  |  |  |  |  |  |  |
| Vastus Lateralis Thickness (cm) |  | 2.54 ± 0.32 |  | 2.75 ± 0.30 |  | **<0.05** |  | 2.6 ± 0.42 |  | 2.96 ± 0.41 |  | **<0.05** |
| Rectus Femoris + Vastus Intermedius Thickness (cm) |  | 4.34 ± 0.73 |  | 4.98 ± 0.84 |  | **<0.05** |  | 4.26 ± 0.75 |  | 4.90 ± 0.75 |  | **<0.05** |
| Total Muscle Thickness (cm) |  | 6.88 ± 0.86 |  | 7.74 ± 0.96 |  | **<0.05** |  | 7.00 ± 1.00 |  | 7.86 ± 1.01 |  | **<0.05** |
|  |  |  |  |  |  |  |  |  |  |  |  |  |
| **Volume** |  |  |  |  |  |  |  |  |  |  |  |  |
| Leg Press Training Session Total Volume (au) |  | 5474 ± 2077 |  | 7775 ± 2147 |  | **<0.05** |  | 4720 ± 2060 |  | 7739 ± 2510 |  | **<0.05** |
| Leg Extension Training Session Total volume (au) |  | 1247 ± 378 |  | 1948 ± 490 |  | **<0.05** |  | 1237 ± 338 |  | 1808 ± 218 |  | **<0.05** |

^Physiological adaptations to 8 weeks of resistance training under low-load (LL) and high-load (HL) conditions. Data are presented as mean ± standard deviation for pre- and post-training values. Strength outcomes include one-repetition maximum (1RM) for leg press and leg extension. Muscle hypertrophy was assessed via ultrasound-measured thickness of the vastus lateralis, rectus femoris + vastus intermedius, and total anterior thigh. Training volume is expressed in arbitrary units (au) as the cumulative load (sets × repetitions × weight) per session for leg press and leg extension exercises. p &lt; 0.05 indicates statistically significant within-group differences (Before vs. After).^

**Table S3** – Performance of Random Forest classification models distinguishing three groups (Before, After-LL, and After-HL) using different metabolite subsets.

| **Model** |  | **Accuracy** |  | **F1-score Before** |  | **F1-score After-LL** |  | **F1-score After-HL** |  |  | **Metabolites (Feature Importance)** | | | | |
| --- | --- | --- | --- | --- | --- | --- | --- | --- | --- | --- | --- | --- | --- | --- | --- |
| **All 48 metabolites** |  | 0.62 |  | 0.83 |  | 0.29 |  | 0.44 |  |  | Acetone (0.117) | 3-Hydroxyisovalerate (0.113) | 3-Hydroxybutyrate (0.042) | 4-Pyridoxate (0.039) | 2-Hydroxyisovalerate (0.034) |
|  |  |  |  |  |  |  |  |  |  |  | Isobutyrate (0.033) | Inosine (0.028) | Isoleucine (0.027) | Acetoacetate (0.026) | 2-Oxoisocaproate (0.022) |
|  |  |  |  |  |  |  |  |  |  |  | Hypoxanthine (0.022) | Lactate (0.021) | Methylguanidine (0.021) | Pantothenate (0.018) | Methylsuccinate (0.016) |
|  |  |  |  |  |  |  |  |  |  |  | Pyruvate (0.016) | Creatinine (0.016) | Tyrosine (0.016) | Glucose (0.015) | O-Phosphocholine (0.015) |
|  |  |  |  |  |  |  |  |  |  |  | Leucine (0.015) | Phenylalanine (0.015) | Citrate (0.015) | Malonate (0.014) | Thymidine (0.012) |
|  |  |  |  |  |  |  |  |  |  |  | 2-Hydroxybutyrate (0.012) | 3-Methyl-2-oxovalerate (0.011) | 3-Hydroxyisobutyrate (0.011) | Ornithine (0.011) | Glycerol (0.011) |
|  |  |  |  |  |  |  |  |  |  |  | Valine (0.010) | Threonine (0.010) | Alanine (0.010) | Choline (0.009) | 2-Aminobutyrate (0.009) |
|  |  |  |  |  |  |  |  |  |  |  | Methylamine (0.008) | Trimethylamine (0.008) | Lysine (0.008) | Proline (0.008) | Valerate (0.007) |
|  |  |  |  |  |  |  |  |  |  |  | Glutamine (0.006) | O-Acetylcarnitine (0.006) | N-Methylhydantoin (0.006) | Dimethyl sulfone (0.006) | Oxypurinol (0.005) |
|  |  |  |  |  |  |  |  |  |  |  | Glycine (0.005) | Succinate (0.005) | Asparagine (0.004) |  |  |
|  |  |  |  |  |  |  |  |  |  |  |  |  |  |  |  |
| **1 metabolite** |  | 0.76 |  | 0.97 |  | 0.50 |  | 0.59 |  |  | 3-Hydroxyisovalerate (1.000) |  |  |  |  |
|  |  |  |  |  |  |  |  |  |  |  |  |  |  |  |  |
| **2 metabolites** |  | 0.82 |  | 0.94 |  | 0.62 |  | 0.74 |  |  | 3-Hydroxyisovalerate (0.637) | 2-Hydroxyisovalerate (0.363) |  |  |  |
|  |  |  |  | 1.00 |  | 0.63 |  | 0.67 |  |  | 3-Hydroxyisovalerate (0.794) | Dimethyl sulfone (0.205) |  |  |  |
| **3 metabolites** |  | 0.85 |  | 0.88 |  | 0.71 |  | 0.94 |  |  | Acetone (0.463) | Glucose (0.291) | 3-Hydroxyisobutyrate (0.244) |  |  |
|  |  |  |  | 0.92 |  | 0.71 |  | 0.82 |  |  | 3-Hydroxyisovalerate (0.473) | 4-Pyridoxate (0.296) | Glucose (0.229) |  |  |
|  |  |  |  | 0.94 |  | 0.75 |  | 0.75 |  |  | 3-Hydroxyisovalerate (0.516) | Choline (0.244) | Glucose (0.238) |  |  |
|  |  |  |  | 1.00 |  | 0.67 |  | 0.74 |  |  | 3-Hydroxyisovalerate (0.500) | Glucose (0.264) | O-Acetylcarnitine (0.234) |  |  |
|  |  |  |  | 0.97 |  | 0.75 |  | 0.71 |  |  | 3-Hydroxyisovalerate (0.482) | Glucose (0.285) | Valine (0.232) |  |  |
|  |  |  |  | 0.97 |  | 0.75 |  | 0.71 |  |  | 3-Hydroxyisovalerate (0.511) | Malonate (0.256) | O-Phosphocholine (0.231) |  |  |
| **4 metabolites** |  | 0.91 |  | 0.97 |  | 0.80 |  | 0.90 |  |  | 3-Hydroxyisovalerate (0.432) | Acetone (0.322) | O-Phosphocholine (0.147) | Dimethyl sulfone (0.097) |  |
|  |  |  |  |  |  |  |  |  |  |  |  |  |  |  |  |
| **5 metabolites** |  | 0.91 |  | 1.00 |  | 0.80 |  | 0.84 |  |  | 3-Hydroxyisovalerate (0.406) | 4-Pyridoxate (0.194) | Isoleucine (0.160) | Acetoacetate (0.132) | O-Acetylcarnitine (0.106) |
|  |  |  |  | 0.97 |  | 0.82 |  | 0.89 |  |  | 3-Hydroxyisovalerate (0.397) | Isobutyrate (0.204) | 4-Pyridoxate (0.198) | O-Phosphocholine (0.113) | Alanine (0.086) |
|  |  |  |  | 1.00 |  | 0.82 |  | 0.82 |  |  | 3-Hydroxyisovalerate (0.397) | 4-Pyridoxate (0.186) | Isobutyrate (0.178) | Glycerol (0.119) | O-Phosphocholine (0.117) |
|  |  |  |  | 0.97 |  | 0.80 |  | 0.89 |  |  | 3-Hydroxyisovalerate (0.444) | Acetoacetate (0.176) | Hypoxanthine (0.168) | O-Acetylcarnitine (0.127) | Asparagine (0.083) |
|  |  |  |  | 0.97 |  | 0.86 |  | 0.84 |  |  | 3-Hydroxyisovalerate (0.480) | Glucose (0.151) | Choline (0.133) | Proline (0.128) | Glutamine (0.105) |
|  |  |  |  | 0.97 |  | 0.86 |  | 0.84 |  |  | 3-Hydroxyisovalerate (0.481) | Glucose (0.173) | Choline (0.153) | Glycerol (0.132) | Valerate (0.058) |
|  |  |  |  | 0.97 |  | 0.88 |  | 0.82 |  |  | 3-Hydroxyisovalerate (0.466) | Glucose (0.165) | Phenylalanine (0.162) | Choline (0.155) | Valerate (0.050) |
|  |  |  |  | 1.00 |  | 0.82 |  | 0.82 |  |  | 3-Hydroxyisovalerate (0.482) | Glucose (0.154) | Choline (0.141) | Proline (0.135) | Succinate (0.085) |
|  |  |  |  | 1.00 |  | 0.82 |  | 0.82 |  |  | 3-Hydroxyisovalerate (0.485) | Glucose (0.170) | Choline (0.146) | Proline (0.141) | Valerate (0.055) |
|  |  |  |  | 0.87 |  | 0.86 |  | 0.84 |  |  | 3-Hydroxyisovalerate (0.443) | Glucose (0.170) | Methylguanidine (0.141) | Proline (0.129) | O-Phosphocholine (0.115) |
|  |  |  |  | 0.97 |  | 0.80 |  | 0.89 |  |  | 3-Hydroxyisovalerate (0.463) | Isobutyrate (0.177) | Hypoxanthine (0.146) | O-Acetylcarnitine (0.107) | O-Phosphocholine (0.105) |

^Performance metrics of Random Forest classification models distinguishing the three experimental groups (Before, After-LL, and After-HL) based on serum metabolomic profiles. Models were trained using different metabolite subsets, ranging from the full set of 48 metabolites to optimized minimal panels containing 1 to 5 metabolites. Accuracy and F1-scores are reported for each group. Feature importance scores (in parentheses) indicate the relative contribution of each metabolite to the classification performance. All 48 metabolites were assigned MSI Level 2 confidence in accordance with the Metabolomics Standards Initiative (MSI).^

**Table S4** – Univariate analysis of serum metabolites: within-protocol paired t-test and three-group one-way ANOVA.

| **Metabolite** |  | **Paired t-test (within-protocol: PRE vs POST)** | | | | | | | | |  | **One-way ANOVA (three-group: Before, After-LL, After-HL)** | | | | | | | | | |
| --- | --- | --- | --- | --- | --- | --- | --- | --- | --- | --- | --- | --- | --- | --- | --- | --- | --- | --- | --- | --- | --- |
|  |  | **Low-load (LL)** | | | |  | **High-load (HL)** | | | |  | **Omnibus** | |  | **Post-hoc: After-LL vs Before** | | |  | **Post-hoc: After-HL vs Before** | | |
|  |  | **p** | **p (FDR)** | **Δ** | **Fold (POS/PRE)** |  | **p** | **p (FDR)** | **Δ** | **Fold (POS/PRE)** |  | **p** | **p (FDR)** |  | **p** | **Δ** | **Fold** |  | **p** | **Δ** | **Fold** |
| 2-Aminobutyrate |  | 0.058 | 0.324 | ↑ | 1.127 |  | 0.037 | 0.285 | ↑ | 1.142 |  | 0.044 | 0.236 |  | 0.349 | ↑ | 1.072 |  | 0.013 | ↑ | 1.192 |
| 2-Hydroxybutyrate |  | 0.59 | 0.802 | ↑ | 1.221 |  | 0.019 | 0.181 | ↑ | 2.446 |  | 0.038 | 0.23 |  | 0.35 | ↑ | 1.408 |  | 0.011 | ↑ | 2.112 |
| 2-Hydroxyisovalerate |  | 0.259 | 0.634 | ↑ | 1.267 |  | 0.005 | 0.085 | ↑ | 1.451 |  | 0.037 | 0.23 |  | 0.184 | ↑ | 1.249 |  | 0.012 | ↑ | 1.469 |
| 2-Oxoisocaproate |  | 0.601 | 0.802 | ↓ | 0.814 |  | 0.347 | 0.666 | ↑ | 1.253 |  | 0.145 | 0.521 |  | 0.343 | ↓ | 0.791 |  | 0.182 | ↑ | 1.285 |
| 3-Hydroxybutyrate |  | 0.035 | 0.324 | ↑ | 1.286 |  | 0.016 | 0.181 | ↑ | 1.392 |  | 0.01 | 0.12 |  | 0.015 | ↑ | 1.335 |  | 0.01 | ↑ | 1.345 |
| 3-Hydroxyisobutyrate |  | 0.377 | 0.725 | ↑ | 2.185 |  | 0.219 | 0.501 | ↑ | 2.226 |  | 0.212 | 0.597 |  | 0.383 | ↑ | 1.81 |  | 0.085 | ↑ | 2.567 |
| 3-Hydroxyisovalerate |  | <0.001 | 0.004 | ↑ | 3.003 |  | <0.001 | <0.001 | ↑ | 6.721 |  | <0.001 | <0.001 |  | <0.001 | ↑ | 4.129 |  | <0.001 | ↑ | 4.483 |
| 3-Methyl-2-oxovalerate |  | 0.061 | 0.324 | ↑ | 1.52 |  | 0.172 | 0.482 | ↑ | 1.31 |  | 0.068 | 0.298 |  | 0.059 | ↑ | 1.438 |  | 0.074 | ↑ | 1.373 |
| 4-Pyridoxate |  | 0.12 | 0.522 | ↓ | 0.912 |  | 0.191 | 0.482 | ↓ | 0.927 |  | 0.183 | 0.549 |  | 0.301 | ↓ | 0.941 |  | 0.076 | ↓ | 0.901 |
| Acetoacetate |  | 0.232 | 0.618 | ↓ | 0.917 |  | 0.104 | 0.383 | ↑ | 1.295 |  | 0.017 | 0.162 |  | 0.493 | ↓ | 0.917 |  | 0.015 | ↑ | 1.295 |
| Acetone |  | 0.013 | 0.152 | ↑ | 1.693 |  | <0.001 | <0.001 | ↑ | 2.286 |  | <0.001 | <0.001 |  | <0.001 | ↑ | 1.94 |  | <0.001 | ↑ | 1.99 |
| Alanine |  | 0.601 | 0.802 | ↑ | 1.06 |  | 0.861 | 0.921 | ↓ | 0.99 |  | 0.947 | 0.967 |  | 0.813 | ↑ | 1.02 |  | 0.769 | ↑ | 1.023 |
| Asparagine |  | 0.329 | 0.717 | ↓ | 0.845 |  | 0.508 | 0.747 | ↓ | 0.909 |  | 0.651 | 0.842 |  | 0.587 | ↓ | 0.905 |  | 0.378 | ↓ | 0.852 |
| Choline |  | 0.475 | 0.761 | ↓ | 0.939 |  | 0.903 | 0.922 | ↓ | 0.978 |  | 0.575 | 0.842 |  | 0.797 | ↑ | 1.033 |  | 0.383 | ↓ | 0.891 |
| Citrate |  | 0.371 | 0.725 | ↓ | 0.951 |  | 0.514 | 0.747 | ↓ | 0.962 |  | 0.251 | 0.635 |  | 0.137 | ↓ | 0.894 |  | 0.847 | ↑ | 1.013 |
| Creatinine |  | 0.599 | 0.802 | ↑ | 1.013 |  | 0.262 | 0.573 | ↑ | 1.057 |  | 0.355 | 0.736 |  | 0.917 | ↓ | 0.994 |  | 0.19 | ↑ | 1.075 |
| Dimethyl sulfone |  | 1 | 1 | = | 1 |  | 0.837 | 0.921 | ↑ | 1.052 |  | 0.851 | 0.95 |  | 0.837 | ↓ | 0.951 |  | 0.669 | ↑ | 1.097 |
| Glucose |  | 0.809 | 0.958 | ↓ | 0.97 |  | 0.398 | 0.693 | ↓ | 0.938 |  | 0.47 | 0.777 |  | 0.235 | ↓ | 0.91 |  | 0.887 | ↓ | 0.99 |
| Glutamine |  | 0.451 | 0.761 | ↓ | 0.957 |  | 0.785 | 0.919 | ↑ | 1.014 |  | 0.935 | 0.967 |  | 0.762 | ↓ | 0.985 |  | 0.772 | ↓ | 0.986 |
| Glycerol |  | 0.98 | 1 | ↑ | 1.004 |  | 0.482 | 0.746 | ↑ | 1.085 |  | 0.553 | 0.842 |  | 0.894 | ↓ | 0.985 |  | 0.333 | ↑ | 1.103 |
| Glycine |  | 0.464 | 0.761 | ↓ | 0.949 |  | 0.131 | 0.448 | ↓ | 0.891 |  | 0.44 | 0.777 |  | 0.255 | ↓ | 0.906 |  | 0.355 | ↓ | 0.927 |
| Hypoxanthine |  | 0.47 | 0.761 | ↓ | 0.874 |  | 0.419 | 0.693 | ↓ | 0.882 |  | 0.646 | 0.842 |  | 0.505 | ↓ | 0.89 |  | 0.405 | ↓ | 0.868 |
| Inosine |  | 0.104 | 0.501 | ↑ | 1.229 |  | 0.098 | 0.383 | ↑ | 1.248 |  | 0.061 | 0.294 |  | 0.692 | ↑ | 1.067 |  | 0.069 | ↑ | 1.394 |
| Isobutyrate |  | 0.01 | 0.152 | ↑ | 1.488 |  | 0.042 | 0.285 | ↑ | 1.721 |  | 0.005 | 0.075 |  | 0.344 | ↑ | 1.266 |  | 0.001 | ↑ | 1.95 |
| Isoleucine |  | 0.006 | 0.135 | ↑ | 1.209 |  | 0.101 | 0.383 | ↑ | 1.113 |  | 0.02 | 0.162 |  | 0.175 | ↑ | 1.099 |  | 0.006 | ↑ | 1.203 |
| Lactate |  | 0.041 | 0.324 | ↓ | 0.885 |  | 0.041 | 0.693 | ↓ | 0.921 |  | 0.635 | 0.842 |  | 0.377 | ↓ | 0.884 |  | 0.537 | ↓ | 0.922 |
| Leucine |  | 0.507 | 0.786 | ↓ | 0.878 |  | 0.05 | 0.303 | ↓ | 0.643 |  | 0.118 | 0.472 |  | 0.626 | ↓ | 0.909 |  | 0.078 | ↓ | 0.622 |
| Lysine |  | 0.771 | 0.958 | ↓ | 0.982 |  | 0.315 | 0.63 | ↑ | 1.116 |  | 0.644 | 0.842 |  | 0.832 | ↑ | 1.019 |  | 0.355 | ↑ | 1.079 |
| Malonate |  | 0.049 | 0.324 | ↓ | 0.748 |  | 0.14 | 0.448 | ↑ | 1.263 |  | 0.485 | 0.777 |  | 0.407 | ↓ | 0.868 |  | 0.585 | ↑ | 1.083 |
| Methylamine |  | 0.802 | 0.958 | ↓ | 0.821 |  | 0.284 | 0.592 | ↑ | 1.692 |  | 0.486 | 0.777 |  | 0.857 | ↓ | 0.905 |  | 0.291 | ↑ | 1.539 |
| Methylguanidine |  | 0.458 | 0.761 | ↓ | 0.834 |  | 0.201 | 0.482 | ↓ | 0.725 |  | 0.291 | 0.699 |  | 0.674 | ↓ | 0.908 |  | 0.12 | ↓ | 0.668 |
| Methylsuccinate |  | 0.205 | 0.597 | ↑ | 1.126 |  | 0.066 | 0.319 | ↑ | 1.114 |  | 0.156 | 0.521 |  | 0.202 | ↑ | 1.1 |  | 0.074 | ↑ | 1.137 |
| N-Methylhydantoin |  | 0.922 | 0.967 | ↓ | 0.993 |  | 0.627 | 0.809 | ↑ | 1.102 |  | 0.935 | 0.967 |  | 0.742 | ↑ | 1.055 |  | 0.796 | ↑ | 1.041 |
| O-Acetylcarnitine |  | 0.818 | 0.958 | ↓ | 0.978 |  | 0.6 | 0.809 | ↓ | 0.938 |  | 0.84 | 0.95 |  | 0.714 | ↓ | 0.965 |  | 0.579 | ↓ | 0.949 |
| O-Phosphocholine |  | 0.165 | 0.528 | ↑ | 1.191 |  | 0.893 | 0.922 | ↓ | 0.966 |  | 0.383 | 0.736 |  | 0.255 | ↑ | 1.247 |  | 0.719 | ↓ | 0.926 |
| Ornithine |  | 0.268 | 0.634 | ↓ | 0.954 |  | 0.942 | 0.942 | ↑ | 1.005 |  | 0.673 | 0.842 |  | 0.468 | ↓ | 0.939 |  | 0.812 | ↑ | 1.019 |
| Oxypurinol |  | 0.894 | 0.967 | ↑ | 1.038 |  | 0.777 | 0.919 | ↑ | 1.099 |  | 0.815 | 0.95 |  | 0.59 | ↑ | 1.194 |  | 0.889 | ↓ | 0.952 |
| Pantothenate |  | 0.139 | 0.528 | ↑ | 1.146 |  | 0.55 | 0.776 | ↑ | 1.07 |  | 0.39 | 0.736 |  | 0.183 | ↑ | 1.142 |  | 0.467 | ↑ | 1.074 |
| Phenylalanine |  | 0.211 | 0.597 | ↑ | 1.039 |  | 0.062 | 0.319 | ↑ | 1.109 |  | 0.163 | 0.521 |  | 0.442 | ↑ | 1.043 |  | 0.059 | ↑ | 1.105 |
| Proline |  | 0.927 | 0.967 | ↓ | 0.993 |  | 0.678 | 0.835 | ↓ | 0.959 |  | 0.248 | 0.635 |  | 0.201 | ↓ | 0.886 |  | 0.551 | ↑ | 1.05 |
| Pyruvate |  | 0.165 | 0.528 | ↓ | 0.845 |  | 0.479 | 0.746 | ↓ | 0.877 |  | 0.334 | 0.736 |  | 0.15 | ↓ | 0.748 |  | 0.835 | ↓ | 0.965 |
| Succinate |  | 0.921 | 0.967 | ↑ | 1.006 |  | 0.863 | 0.921 | ↓ | 0.972 |  | 0.979 | 0.979 |  | 0.84 | ↓ | 0.978 |  | 0.97 | ↓ | 0.996 |
| Threonine |  | 0.68 | 0.882 | ↑ | 1.055 |  | 0.373 | 0.688 | ↓ | 0.928 |  | 0.684 | 0.842 |  | 0.501 | ↓ | 0.921 |  | 0.77 | ↑ | 1.033 |
| Thymidine |  | 0.871 | 0.967 | ↓ | 0.949 |  | 0.165 | 0.482 | ↓ | 0.785 |  | 0.701 | 0.842 |  | 0.758 | ↓ | 0.925 |  | 0.404 | ↓ | 0.803 |
| Trimethylamine |  | 0.55 | 0.802 | ↓ | 0.941 |  | 0.621 | 0.809 | ↓ | 0.952 |  | 0.88 | 0.96 |  | 0.672 | ↓ | 0.944 |  | 0.692 | ↓ | 0.949 |
| Tyrosine |  | 0.277 | 0.634 | ↑ | 1.085 |  | 0.192 | 0.482 | ↑ | 1.09 |  | 0.398 | 0.736 |  | 0.235 | ↑ | 1.097 |  | 0.315 | ↑ | 1.079 |
| Valerate |  | 0.152 | 0.528 | ↑ | 1.057 |  | 0.853 | 0.921 | ↑ | 1.01 |  | 0.611 | 0.842 |  | 0.38 | ↑ | 1.036 |  | 0.473 | ↑ | 1.028 |
| Valine |  | 0.349 | 0.725 | ↑ | 1.006 |  | 0.64 | 0.809 | ↑ | 1.002 |  | 0.362 | 0.736 |  | 0.998 | ↑ | 1 |  | 0.181 | ↑ | 1.008 |

^Serum metabolite concentrations were compared within each protocol by paired t-test (PRE vs POST) and across the three experimental groups (Before, After-LL, and After-HL) by one-way ANOVA, followed by post-hoc comparisons of each post-training group against Before. LL: low-load; HL: high-load; PRE: pre-training; POST: post-training; p: unadjusted p-value; p (FDR): p-value adjusted for the false discovery rate; Δ: direction of change (↑ increase, ↓ decrease, = no change); Fold: ratio of group means (for the paired t-test, the POST/PRE ratio; values < 1 indicate a decrease). All 48 metabolites were assigned MSI Level 2 confidence in accordance with the Metabolomics Standards Initiative (MSI).^

**Supplementary Figures**

**Fig. S1** Multivariate analyses and classification performance of serum metabolomic profiles for the three study groups (Before, After-LL, and After-HL). (A) Principal Component Analysis (PCA) and (B) Partial Least Squares Discriminant Analysis (PLS-DA) score plots based on all quantified metabolites. (C) F1-scores from Random Forest models constructed with subsets of 1– 5 metabolites compared with the full panel (48 metabolites). Ellipses represent the 95 % confidence regions for each class (green = Before, blue = After-LL, red = After-HL).

**Fig. S2** Volcano plots of serum metabolite changes following After-LL (A) and After-HL (B) resistance training compared to Before. Blue points represent upregulated metabolites and red points represent downregulated metabolites.

**Fig. S3** Multivariate analyses and classification performance of serum metabolomic profiles before and after 8 weeks of resistance training. (A) Principal Component Analysis (PCA) and (B) Partial Least Squares Discriminant Analysis (PLS-DA) score plots based on all quantified metabolites. Ellipses represent the 95 % confidence regions for each class (green = Before; purple = After). (C) F1-scores of Random Forest models constructed using subsets of one to three metabolites and the full 48-metabolite panel.

**Fig. S4** Permutation plots for top-performing models of two groups (Before, After). (A) 3_Hydroxyisovalerate + Acetoacetate (B) 3_Hydroxyisovalerate + Choline (C) 3_Hydroxyisovalerate + Oxypurinol. Red line = Real model; Blue bars = permuted models.

**Fig. S5** Multivariate analyses of serum metabolomic profiles for two groups (After LL, After HL). (A) PCA and (B) PLS-DA score plots based on all metabolites. (C) F1-scores of Random Forest models constructed using subsets of 1–3 metabolites compared to the full panel (48 metabolites).

**Figure S6** – Permutation plots for top-performing model of two groups (After-LL, After-HL). Choline + Glucose + Alanine. Red line = Real model; Blue bars = permuted models.

**References**

Breiman, L. (2001). Random Forests. *Machine Learning*, *45*(1), 5–32. https://doi.org/10.1023/A:1010933404324

Broadhurst, D. I., & Kell, D. B. (2007). Statistical strategies for avoiding false discoveries in metabolomics and related experiments. *Metabolomics*, *2*(4), 171–196. https://doi.org/10.1007/s11306-006-0037-z

Brown L.E., W. J. (2001). ASEP Procedures recommendation I: Accurate assessment of muscular strength and power. *J. Exerc. Physiol. Online*, *4*, 1–21.

Dona, A. C., Jiménez, B., Schäfer, H., Humpfer, E., Spraul, M., Lewis, M. R., et al. (2014). Precision High-Throughput Proton NMR Spectroscopy of Human Urine, Serum, and Plasma for Large-Scale Metabolic Phenotyping. *Analytical Chemistry*, *86*(19), 9887–9894. https://doi.org/10.1021/ac5025039

Hinojosa Lee, M. C., Braet, J., & Springael, J. (2024). Performance Metrics for Multilabel Emotion Classification: Comparing Micro, Macro, and Weighted F1-Scores. *Applied Sciences*, *14*(21), 9863. https://doi.org/10.3390/app14219863

Nam, S. L., de la Mata, A. P., Dias, R. P., & Harynuk, J. J. (2020). Towards Standardization of Data Normalization Strategies to Improve Urinary Metabolomics Studies by GC×GC-TOFMS. *Metabolites*, *10*(9), 376. https://doi.org/10.3390/metabo10090376

Pérez-Guaita, D., Kuligowski, J., Garrigues, S., Quintás, G., & Wood, B. R. (2015). Assessment of the statistical significance of classifications in infrared spectroscopy based diagnostic models. *The Analyst*, *140*(7), 2422–2427. https://doi.org/10.1039/C4AN01783H

Rosen Vollmar, A. K., Rattray, N. J. W., Cai, Y., Santos-Neto, Á. J., Deziel, N. C., Jukic, A. M. Z., & Johnson, C. H. (2019). Normalizing Untargeted Periconceptional Urinary Metabolomics Data: A Comparison of Approaches. *Metabolites*, *9*(10), 198. https://doi.org/10.3390/metabo9100198

Shimizu, A., Ikeguchi, M., & Sugai, S. (1994). Appropriateness of DSS and TSP as internal references for 1H NMR studies of molten globule proteins in aqueous media. *Journal of Biomolecular NMR*, *4*(6), 859–862. https://doi.org/10.1007/BF00398414

Wishart, D. S., Feunang, Y. D., Marcu, A., Guo, A. C., Liang, K., Vázquez-Fresno, R., et al. (2018). HMDB 4.0: the human metabolome database for 2018. *Nucleic Acids Research*, *46*(D1), D608–D617. https://doi.org/10.1093/nar/gkx1089
